# ARHGAP39 plays an essential role in anxiety-like behavior and stress response

**DOI:** 10.1038/s41398-026-04088-1

**Published:** 2026-05-09

**Authors:** Shin-Meng Deng, Yu-Ju Chen, Wei-Chien Hung, Kuan-Yu Wu, Wei-Che Chang, Irene Han-Juo Cheng, Mau-Sun Chang, Guo-Jen Huang

**Affiliations:** 1 Graduate Institute of Biomedical Sciences, College of Medicine, Chang Gung University, Taoyuan, Taiwan; 2 Institute of Brain Science, College of Medicine, National Yang Ming Chiao Tung University, Taipei, Taiwan; 3 Institute of Biochemical Sciences, National Taiwan University, Taipei, Taiwan; 4 Department of Biomedical Sciences, College of Medicine, Chang Gung University, Taoyuan, Taiwan; 5 Neuroscience Research Center, Chang Gung Memorial Hospital at Linkou, Taoyuan, Taiwan

**Keywords:** Neuroscience, Physiology

## Abstract

Anxiety is a common mental health disorder marked by heightened stress responses and impaired emotional regulation. While the hippocampus and the hypothalamic–pituitary–adrenal (HPA) axis play key roles in regulating anxiety, the molecular mechanisms remain incompletely understood. In this study, we identified a strong link between ARHGAP39 function and anxiety using brain-specific *Arhgap39* conditional knockout (cKO) mice. ARHGAP39 is a brain-enriched Rho GTPase-activating protein implicated in neurodevelopment and synaptic regulation. *Arhgap39* cKO mice exhibited ectopic progenitor cells in the hilus of the hippocampus during early postnatal development, along with impaired adult neurogenesis in dentate gyrus (DG). Behaviorally, adult cKO mice demonstrated pronounced anxiety-like phenotypes without cognitive impairments, whereas aged cKO mice exhibited persistent anxiety accompanied by learning and memory deficits. Molecular profiling revealed that cKO mice exhibited significantly elevated RNA expression levels of glutamatergic and glucocorticoid receptor-related genes in the hippocampus. In addition, cKO mice exhibited more stress-activated neurons in the DG. Mechanistically, *Arhgap39* deficiency disrupted Rho GTPase–related actin pathways, leading to elevated Cofilin-1 and Arp3 expression and reduced PSD-95 levels, suggesting disruption of synaptic organization. Finally, hippocampal overexpression of *Arhgap39* in C57BL/6 mice did not affect baseline behavior but alleviated chronic stress-induced anxiety in light-dark box test. In conclusion, our findings demonstrate that ARHGAP39 plays a critical role in modulating anxiety, hippocampal neurogenesis, and synaptic stability, highlighting it as a promising target for developing anxiolytic therapies.

## Introduction

ARHGAP39, also known as Vilse or Porf-2, is a Rho family GTPase-activating protein (RhoGAP) that is highly conserved across species, from *Drosophila* to mammals [1]. It plays a fundamental role in cytoskeletal dynamics, synaptic plasticity, and neural development [2–9]. Early studies identified ARHGAP39 as a hypothalamic gene involved in reproductive regulation [10–15], while in *Drosophila*, it was shown to mediate axonal guidance and midline repulsion, emphasizing its role in neural connectivity [1, 16]. In mammals, ARHGAP39 is highly expressed in the central nervous system, where it regulates axon growth, branching, and neural stem cell proliferation through the Wnt/β-catenin pathway [4, 6].

Recent genetic studies have linked ARHGAP39 mutations to severe neurodevelopmental disorders in humans. A homozygous variant in ARHGAP39 (c.1301 G > T; p.Cys434Phe) was identified in a consanguineous Saudi family, where affected individuals exhibited lethal cerebellar vermis hypoplasia, facial dysmorphology, and visual impairments [9]. This study highlights the critical role of ARHGAP39 in normal cerebellar and craniofacial development in humans. Furthermore, ARHGAP39 has been associated with hippocampal spine morphogenesis, through its interaction with CNKSR2, a synaptic scaffold protein that regulates Ras/MAPK signaling [3, 8]. Altogether, ARHGAP39 dysfunction contributes to a spectrum of human neurodevelopmental disorders.

Conditional knockout (cKO) mouse models have provided further insights into the role of ARHGAP39, which is highly expressed in the hippocampus, as shown in the Allen Brain Atlas. Loss of ARHGAP39 leads to dendritic abnormalities in the hippocampus, reduced spine density, and impaired long-term potentiation (LTP), ultimately resulting in hippocampus-dependent learning and memory deficits [5]. Although the hippocampus plays a key role in both cognitive function and emotional regulation [17], the involvement of ARHGAP39 in emotion and stress has yet to be investigated. Given that, we sought to investigate the role of ARHGAP39 in anxiety and stress responses using brain-specific *Arhgap39* cKO mice.

In the study, we found that ARHGAP39 deficiency affected adult hippocampal neurogenesis, disrupted synaptic stability, and increased anxiety levels. Conversely, overexpression of ARHGAP39 in the hippocampus alleviated some, but not all, anxiety-like behaviors in C57BL/6 mice under chronic stress, suggesting its potential as a therapeutic target for stress-related disorders.

## Materials and methods

### Animals

All animal experiments were conducted using WT and *Arhgap39* cKO mice at P10, adulthood (8 to 12-week-old), and aging (18-month-old). *Arhgap39* cKO mice were generated by crossing *Sox1::Cre* mice [18] (Acc. No. [CDB0525K]) with *Arhgap39* floxed mice. The floxed *Arhgap39* line was previously generated and characterized, as reported in earlier study [5]. Male *Arhgap39*^flox/flox^ - *Sox1::Cre* mice were subsequently crossed with female *Arhgap39*^flox/flox^ mice to obtain *Arhgap39*^flox/flox^ - *Sox1::Cre* (cKO) and *Arhgap39*^flox/flox^ (WT) littermates. Mice were maintained in a specific pathogen-free facility, with a 12-h light/dark cycle, at 22–25 °C and 60–70% humidity, with access to food and water.

### AAV preparation and injection

The *Arhgap39* gene was sub-cloned into an AAV vector under the control of *Camk2a* promoter to generate the *Camk2a* -*Arhgap39* construct (VecGene Biotech, Taiwan). For control experiments, an AAV vector encoding *egfp* under the same promoter was used. Viral particles were produced and purified. Stereotaxic injections were performed in adult C57BL/6 mice to deliver the virus bilaterally into both dorsal and ventral part of the hippocampus (5 × 10^11^ viral genomes (vg) / μL for each injection site).

### RNA isolation and quantitative PCR

Total RNA was isolated from dissected hippocampal tissue using TRIzol reagent (Invitrogen, 15596026, CA, USA), followed by phase separation with BCP (Sigma-Aldrich, B9673; MO, USA) according to the manufacturer’s instructions. The RNA pellet was washed, air-dried, and resuspended in DEPC water (ThermoFisher, AM9915G, MA, USA). cDNA was synthesized from 1 μg of total RNA using Modified MMLV Reverse Transcriptase (Protech, PT-MMLV, Taiwan). Quantitative PCR was performed using SYBR Green-based detection (Bio-Rad, 1708882, CA, USA), and relative gene expression levels were calculated using the 2^-ΔΔCt method. GAPDH served as the internal reference gene for normalization. Primer sequences used in this study are listed in Table 1.

**Table 1:** Primers for RT-PCR.

Gene	Forward	Reverse
Arhgap39	CTTTGACAAGCTTGGCTTCC	CCAGTTCTCGATGTCCGTCT
Gria1	GGATACCGGATGCTCTTTCA	GTTGGCGAGGATGTAGTGGT
Grin1	ACTCCCAACGACCACTTCAC	CTGGTGGGAGTAGGGTGGTA
Grin2a	ACGTGACAGAACGCGAACTT	TCAGTGCGGTTCATCAATAACG
Grin2b	GCCATGAACGAGACTGACCC	GCTTCCTGGTCCGTGTCATC
Eaat2	AACAATATGCCCAAGCAGGT	CCAGGATGACACCAAACACA
Eaat3	TGGACCTAAGCATTGGGCAG	CTTCTCTACGATGCCCGTCC
GABA_A_α1	CTACAGCAACCAGCTATACCC	GCTCTCTGTTTAAATACGTGG
GABA_A_β1	ATGATGCATCTGCAGCCA	TGGAGTTCACGTCAGTCA
GABA_A_β2	GCATGTATGTCTGCAGGA	CTGACACCTACTTCCTGA
GABA_A_γ1	TTTCTTACGTGACAGCAATGG	CATGGGAATGAGAGTGGATCC
GABA_A_γ2	GCAATGGATCTCTTTGTA	GTCCATTTTGGCAATGCG
GABA_A_γ3	TGTCGAAAGCCAACCATCAGG	GACTTGCACTCCTCATAGCAG
Crh	AGCCCTTGAATTTCTTGCAG	AGCCCTTGAATTTCTTGCAG
Crhr1	AGCCCTTGAATTTCTTGCAG	CTGCCATCCGGAAGAGGT
Mr	CCGGTATTGGACTTGCTGTT	CAGCCTGAAGTTGGCTCTCT
Gr	AGGCGATACCAGGATTCAGA	GCAAAGCATAGCAGGTTTCC
Gilz	AACACCGAAATGTATCAGACCC	GTTTAACGGAAACCAAATCCCCT
Sgk1	CTGCTCGAAGCACCCTTACC	TCCTGAGGATGGGACATTTTCA
Fkbp5	AACGGAAAGGCGAGGGATAC	ACACCACATCTCGGCAATCA
Gapdh	TGCACCACCAACTGCTTAGC	GGCATGGACTGTGGTCATGAG

### Protein extracts and Western blot analysis

Tissue samples were homogenized in RIPA buffer (BIOTOOLS, TAAR-ZBZ5, Taiwan) supplemented with protease inhibitor cocktail (Sigma-Aldrich, S8830). Lysates were heated at 100 °C for 10 min to denature proteins. Protein concentrations were determined using the Bradford protein assay (Bio-Rad, 5000006). Equal amounts of protein were resolved by SDS–polyacrylamide gel electrophoresis and transferred onto polyvinylidene difluoride (PVDF) membrane (Millipore, IPVH00010, MA, USA). Membranes were blocked in 5% non-fat milk in TBST or Casein blocking buffer (Sigma-Aldrich, B6429) for 1 h at room temperature and incubated overnight at 4 °C with the following primary antibodies: ARHGAP39 (homemade antibody, mouse monoclonal, a.a. 900–1114, as previously described [5]), beta-actin (Sigma-Aldrich, A5441), GFP (Invitrogen, A11122), GAPDH (Santa Cruz, sc-32233, TX, USA), Rock1 (GeneTex, GTX113266, CA, USA), Diaph1 (GeneTex, GTX102057), Arp3 (GeneTex, GTX115345), PSD95 (Cell signaling, #3450, MA, USA), Synaptophysin (Abcam, ab8049, UK), Cofilin-1 (GeneTex, GTX102156), Cdc42 (Cell signaling, #2466), Cdk5 (Millipore, 05364), Pak1 (Santa Cruz, sc-881). After washing with TBST. membranes were incubated for 1 h at room temperature with horseradish peroxidase-conjugated secondary antibody (Bioss, goat anti-rabbit IgG, bs352 0295G-HRP, Bioss; goat anti-mouse IgG, bs-0296G-HRP, Taiwan). Proteins bands were visualized using Immobilon Western Chemiluminescent HRP Substrate (Millipore, WBKLS0500) and detected with the ChemiDoc XRS+ imaging system (Bio-Rad). Band intensities were quantified using ImageJ software (NIH).

### Behavioral tests

All behavioral experiments were video-recorded and analyzed using the EthoVision tracking system (Noldus Information Technology, Netherlands), which automatically generated and exported behavioral data, thereby minimizing experimenter influence. The investigator was not blinded during behavioral testing. Mice used for behavioral analyses were randomized between WT and cKO groups to reduce potential environmental effects.

#### Open field test

One week prior to behavioral testing, mice were subjected to daily handling to minimize stress responses. On the day of testing, mice were habituated to the behavioral testing room for at least 10 min in the presence of the experimenter. Each mouse was gently placed along the wall of a white circular open field arena (radius = 60 cm) and allowed to freely explore the arena for 5 min. The central area was defined as a concentric circle occupying 60% of the total area. A lamp was positioned to illuminate the center of the arena during the test. Behavioral parameters, including total distance traveled, time spent in the central zone, frequency of center entries, and latency to first enter the center, were recorded.

#### Elevated-O-maze

The apparatus consisted of a circular track (radius = 55 cm) elevated 60 cm above the floor, divided into two opposite open arms and two opposite closed arms enclosed by 15 cm high walls. The width of the track was 6 cm. Each mouse was gently placed at the closed arm and allowed to explore the maze freely for 5 min. Behavioral parameters including total distance traveled, time spent in open arms, number of open arm entries, and latency to first enter the open arm were recorded.

#### Light-dark box

The apparatus consisted of two connected compartments: a light chamber and a dark enclosed chamber. The total dimensions were 40 cm × 20 cm × 20 cm, with the light chamber occupying two-thirds of the total area. The light compartment was illuminated using a ceiling-mounted lamp positioned above the center, while the dark compartment remained unlit and covered to prevent light entry. A small opening (5 × 5 cm) allowed free movement between the two chambers. Each mouse was gently placed in the dark compartment and allowed to explore both chambers freely for 5 min. Behavioral parameters including total distance traveled, time spent in the light compartment, number of transitions between compartments were recorded.

#### Y-maze

To assess working memory, mice were subjected to the Y-maze test. The apparatus consisted of three identical arms (each 36 cm long, 5 cm wide, and enclosed by 15 cm high walls) arranged at 120° angles in a Y-shaped configuration. Each mouse was allowed to freely explore all three arms for 8 min. The percentage of spontaneous alternation was calculated using the following formula:

$$({Number}\,{of}\,{alternations})/({Total}\,{number}\,{of}\,{arm}\,{entries}-2)\times 100$$

#### Morris water maze

Spatial learning and memory were assessed using the Morris water maze. The apparatus consisted of a circular pool (diameter = 120 cm) filled with opaque water maintained at 22–24 °C. A hidden circular platform (diameter = 10 cm) was submerged approximately 1 cm below the water surface and placed in a fixed location within one quadrant. Mice were trained to locate the platform using distal visual cues placed around the testing room. Training was conducted over four consecutive days, with each mouse receiving three trials per day. For each trial, the mouse was released from a different starting point along the pool perimeter and allowed 120 s to locate the platform. If the mouse failed to find the platform within the allotted time, it was gently guided to the platform and allowed to rest for 15 s. One week after the completion of training, a probe trial was conducted to assess memory retention. The platform was removed, and mice were allowed to swim freely for 90 s. Behavioral parameters including latency to first platform crossing and the number of platform crossings were recorded.

#### Barnes maze

The apparatus consisted of a circular platform (96 cm in diameter) elevated 60 cm above the floor, with 40 holes (5 cm in diameter) arranged in three concentric circles across the surface. The escape box was placed under a hole located in the outer area of the platform. Bright lighting was used as a mild aversive stimulus to motivate the mice to find the escape hole using visual cues around the testing room. During training, each mouse was placed in the center of the platform and allowed to search for the escape box for up to 120 s. If the mouse did not find the escape hole within the time limit, it was gently guided to it and allowed to stay inside the box for 30 s. Training was performed over 5 consecutive days. A probe trial was carried out 3 days after the last training session. During the probe, the escape box was removed, and the mouse was allowed to explore the platform for 120 s. The latency to reach the target hole and time spent in the target area were recorded. The target area was defined as a fan-shaped region including the target hole and the two neighboring holes on each side.

#### Forced swimming test

Each mouse was individually placed in a transparent cylindrical tank (height = 25 cm, diameter = 20 cm) filled with water maintained at 22–25 °C to a depth of 15 cm. Mice were allowed to swim for 6 min, and the total duration of immobility during the last 4 min was recorded.

#### Tail suspension test

Mice were suspended by the tail using adhesive tape placed approximately 1 cm from the tip of the tail and hung above the surface of a table. Each session lasted for 6 min. The total duration of immobility was recorded.

#### Sucrose preference

Mice were single-housed and habituated to two identical bottles for 2 days, one containing 1% (w/v) sucrose solution and the other containing plain water. Bottle positions were switched daily to avoid side preference. After 24 h of testing, the volumes of consumed sucrose solution and water were measured, and sucrose preference was calculated as follows:

$$\begin{array}{l}Sucrose\,preference{(}{ \% }{)}\\ \,\,{=}{[}sucrose\,intake{/}{(}sucrose\,intake{+}water\,intake{)}{]}{\times }{100}\end{array}$$

All bottles were weighed before and after the test to determine fluid consumption accurately.

### Corticosterone measurement

Blood samples were collected via facial vein puncture at three time points: under basal conditions, following 30 min of restraint stress, and after a 60-min recovery period post-stress. Approximately 100–150 µL of blood was collected into heparin-coated Microvette® capillary tubes (Sarstedt, Microvette CB 300, Germany) and immediately placed on ice. Plasma was separated by centrifugation at 3000 rpm for 15 min at 4 °C and stored at –80 °C until analysis. Plasma corticosterone concentrations were measured using a commercially available enzyme-linked immunosorbent assay (ELISA) kit (Enzo Life Sciences, ADI-900-097, NY, USA), according to the manufacturer’s protocol.

### Statistical analysis

All results are presented as mean ± SEM for each group. Statistical analyses were performed using GraphPad Prism software. Depending on the data distribution and experimental design, comparisons were conducted using analysis of variance (ANOVA) or an unpaired *t*-test. For the data that is not Gaussian distribution, the Mann-Whitney test was used. Statistical significance was defined as *p < 0.05; **p < 0.01; ***p < 0.001. No animals or samples were excluded from the analysis. All collected data were included in the final statistical analyses. The sample size and number of replications were determined based on prior studies in the same research field. Each experiment was repeated at least once or conducted across multiple cohorts of mice. All replication attempts yielded consistent and reproducible results.

### Supplementary methods

For the detailed description of chronic stress paradigm and immunochemistry, see Supplementary Methods and Materials.

## Results

### Presence of ectopic hilar progenitor cells and impaired adult neurogenesis in *Arhgap39* cKO mice

**Fig. 1:**
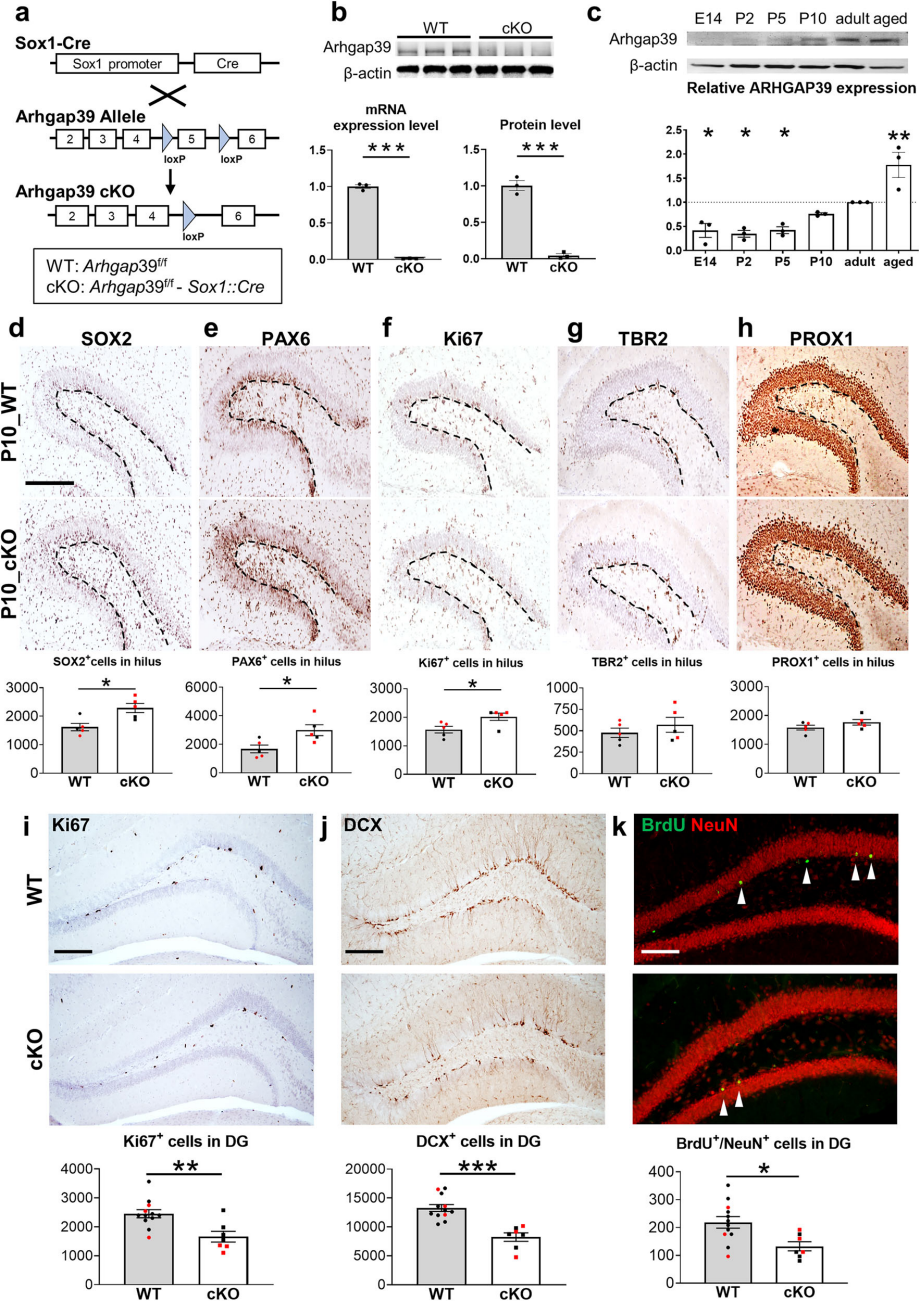
Presence of ectopic hilar progenitor cells and impaired adult neurogenesis in *Arhgap39* cKO Mice. **a** Breeding strategy used to generate *Arhgap39* cKO mice. **b** Hippocampal expression levels of *Arhgap39* were confirmed by RT-qPCR (t_(4)_ = 36.32, p < 0.001) and Western blot analysis (t_(4)_ = 13.22, p < 0.001, N = 3 males per group). **c** ARHGAP39 expression levels were assessed in the hippocampus at multiple developmental stages, including embryonic day 14 (E14), early postnatal stages (P2, P5, P10), adult (P56), and aged (12 months), using Western blot analysis (F_(4,15)_ = 17.75, *p* < 0.001. N = 3 males per group). Dunnett’s multiple comparisons test: E14 (*p* = 0.03), P2 (*p* = 0.015), and P5 (*p* = 0.032), P10 (*p* = 0.567), aged (*p* = 0.004). Data were normalized to the adult group. **d**–**h** Immunohistochemical staining of neuronal proliferation markers in the dentate gyrus (DG) of P10 *Arhgap39* cKO mice and WT littermates. Markers examined include **d** Sox2 (t_(8)_ = 3.244, *p* = 0.011), **e** Pax6 (t_(8)_ = 2.797, *p* = 0.023), **f** Ki67 (U = 3, *p* = 0.047), **g** Tbr2 (t_(8)_ = 0.913, *p* = 0.38), and **h** Prox1 (t_(8)_ = 1.437, *p* = 0.188). Scale bar = 100 μm. WT 2 males, 3 females; cKO 2 males, 3 females. Dashed lines indicate the regions of interest. **i**–**k** Staining of adult neurogenesis markers in the dentate gyrus of *Arhgap39* cKO mice and WT littermates. Markers examined include **i** Ki67 (t_(17)_ = 3.415, *p* = 0.003), **j** Doublecortin (t_(17)_ = 5.243, *p* < 0.001), and **k** BrdU/NeuN (t_(17)_ = 2.849, *p* = 0.011). Scale bar = 100 μm. WT 9 males, 3 females; cKO 4 males, 3 females. Data points in red represent female mice, black represent male mice.

To investigate the role of *Arhgap39* in hippocampal function, we generated brain-specific *Arhgap39* cKO mice by crossing *Sox1::Cre* mice with *Arhgap39* floxed mice, targeting exons 5 of the *Arhgap39* gene (Fig. 1a). In adulthood, we did not observe any difference in either the body or brain size (Fig. S1a, b). To verify the efficiency and specificity of our *Arhgap39* cKO mice, we examined *Arhgap39* expression in various brain regions and peripheral tissues. In the hippocampus, *Arhgap39* was effectively knocked out at both the mRNA and protein levels (Fig. 1b). *Arhgap39* mRNA expression was also significantly reduced in the cerebellum, olfactory bulb, and cerebral cortex. In contrast, peripheral tissues, including the liver, kidney, and muscle, showed no significant changes in *Arhgap39* mRNA expression, indicating brain-specific deletion (Fig. S1c). We further measured ARHGAP39 protein levels in hippocampal tissues across developmental stages and found that it progressively increased with age, suggesting its essential role in age-related developmental processes. (Fig. 1c).

To assess the impact of *Arhgap39* on early postnatal neuronal development and neurogenesis, we examined the DG of wild-type (WT) and cKO mice at postnatal day 10 (P10) and in adulthood. Since hippocampal neurogenesis typically occurs in the subgranular zone of the DG, we analyzed antibody-labeled cells in the hilus to quantify ectopic cell presence. At P10, cKO mice exhibited a significant increase in ectopic progenitor cells in the hilus, marked by elevated SOX2, PAX6, and Ki67 expression, indicating disrupted hippocampal formation (Fig. 1d–f). However, later-stage differentiation markers such as TBR2 and PROX1 returned to WT levels (Fig. 1g, h), suggesting that the developmental dysregulation of the hippocampus is transient.

In adulthood, *Arhgap39* cKO mice exhibited a significant reduction in Ki67-, DCX-, and NeuN/BrdU-positive cells (Fig. 1i–k), with no significant differences in the numbers of Ki67- and DCX-positive cells in the subventricular zone (SVZ) or DCX-positive cells in the olfactory bulb, suggesting that *Arhgap39* deletion specifically affects hippocampal adult neurogenesis (Fig. S2a). Despite this reduction, the intensity and thickness of NeuN-positive cell layers in the DG and CA1 regions remained unchanged (Fig. S2b), suggesting that reduced adult neurogenesis did not result in significant overall neuronal loss. These results highlight the essential role of *Arhgap39* in supporting normal hippocampal development. Moreover, the interneuron microcircuits in the brain have been suggested to modulate the anxiety state [19, 20]. We found that there was no change in the ratio of CTIP2/ PV-positive cells in the subregions of both hippocampus (DG, t_(8)_ = 0.653, *p* = 0.531; CA1, t_(8)_ = 0.946, *p* = 0.371) and cortex (t_(8)_ = 0.476, *p* = 0.646), which rules out the alterations of the excitatory/inhibitory balance in *Arhgap39* cKO mice (Fig. S2c, d).

### Increased anxiety levels without cognitive impairment in adult *Arhgap39* cKO mice

**Fig. 2:**
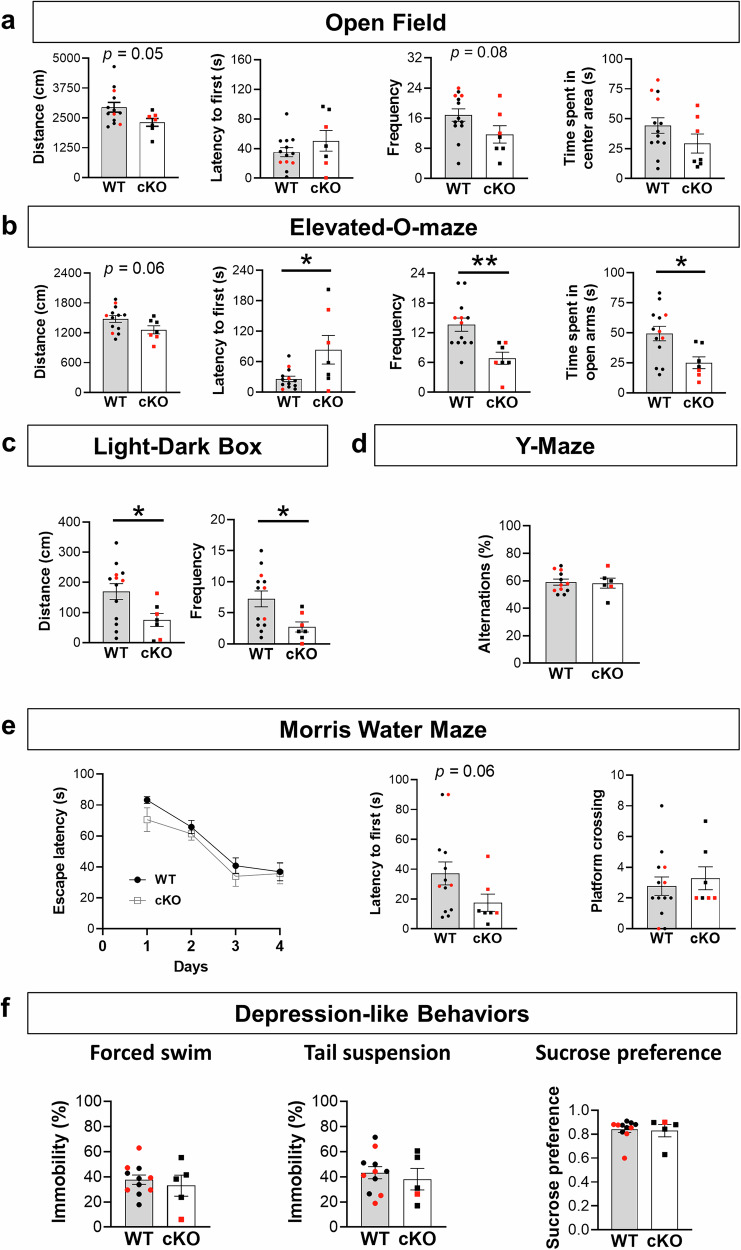
Increased anxiety levels without cognitive impairment in adult *Arhgap39* cKO mice. Performance of adult *Arhgap39* cKO mice in **a** Open Field test: total distance traveled (t_(18)_ = 2.071, *p* = 0.053), latency to first enter the center (t_(18)_ = 1.167, *p* = 0.258), number of center entries (t_(18)_ = 1.834, *p* = 0.083), and time spent in the center zone (t_(18)_ = 1.421, *p* = 0.172) were analyzed. **b** Elevated-O-maze test: total distance traveled (t_(18)_ = 1.834, *p* = 0.083), latency to first enter the open arms (t_(18)_ = 2.674, *p* = 0.015), number of open arm entries (t_(18)_ = 3.337, *p* = 0.003), and time spent in the open arms (t_(18)_ = 2.687, *p* = 0.015) were analyzed. **c** Light-dark box test: distance traveled in lit compartment (t_(18)_ = 2.363, *p* = 0.029) and number of transitions between compartments (t_(18)_ = 2.444, *p* = 0.025) were analyzed. **d** Y-maze: percentage of spontaneous alternation were analyzed (t_(18)_ = 0.188, *p* = 0.853). **e** Morris Water Maze: learning curve during the training sessions (F_(1,18)_ = 1.805, *p* = 0.195), and performance in the probe trial as assessed by latency to first platform crossing (U = 22, *p* = 0.06) and number of platform crossings (U = 39.5, *p* = 0.646). **f** Results of depression-like behavior tests: Forced swim test, percentage of immobility time (t_(14)_ = 0.606, *p* = 0.554); Tail suspension test, percentage of immobility time (t_(14)_ = 0.56, *p* = 0.583); Sucrose preference test, percentage of sucrose preference (t_(14)_ = 0.217, *p* = 0.831). Open field, Elevated-O-maze, Light-dark box, and Water maze: WT 10 males, 3 females, cKO 4 males, 3 females. Y-maze: WT 6 males, 6 females, cKO 4 males, 2 females; depression-like behavior tests: WT 6 males, 5 females, cKO 4 males, 1 female.

Based on the hypothesis that adult neurogenesis is linked to anxiety [21], we assessed the anxiety-like behavior in adult *Arhgap39* cKO mice using the open field test, elevated-O-maze test, and light-dark box test. In the open field test, cKO mice traveled a slightly shorter distance compared to WT mice, though no significant difference was observed in other anxiety-related parameters, including the latency to the first entry into center area, the number of center entries, and the time spent in the center (Fig. 2a). However, in the elevated-O-maze and light-dark box tests, which induce higher levels of stress than the open field test, cKO mice exhibited significantly greater anxiety levels compared to the WT group (Fig. 2b, c). To exclude the possibility that peripheral system defects contributed to the behavioral phenotype, we conducted a battery of sensorimotor tests. The results revealed no significant impairments in cKO mice (Fig. S3a). To further determine whether *Sox1::Cre* expression itself influences behavioral outcomes, we compared *Sox1::Cre* mice and their littermate controls across anxiety-related behavioral tests, sensorimotor function assessments, learning and memory, and measures of adult hippocampal neurogenesis. No significant differences were observed between the two groups (Fig. S4), indicating that Cre expression alone does not contribute to the behavioral phenotypes observed in this study.

Given the behavioral changes, we further assessed glial cell activation in the hippocampus, as it is believed to be one of the underlying mechanisms of anxiety-related behavior [22–25]. To assess glial modulation, we examined astrocyte and microglial expression in the hippocampus. *Arhgap39* cKO mice exhibited stronger GFAP, a marker of astrocytes, intensity in the DG, CA1, and especially CA3, indicating a widespread hyperactivation of astrocytes throughout the hippocampus (Fig. S2e). For the microglial marker Iba-1, it showed enhanced intensity selectively in the CA3, suggesting that microglial activation was localized rather than widespread (Fig. S2f). These results imply that the enhanced astrocytic response in cKO mice may not be due to a classical immune response, but rather the mechanisms associated with *Arhgap39* deficiency.

Furthermore, anxiety-like behavior is often accompanied by cognitive impairments [26, 27], and a previous study suggests that mice lacking *Arhgap39* perform worse in the water maze [5]. We next asked whether *Arhgap39* cKO caused cognition deficiency. In the Y-maze test, which assesses working memory, cKO mice performed comparably to WT mice (Fig. 2d). Similarly, in the Morris water maze, which evaluates spatial learning and memory, cKO mice demonstrated normal learning ability during the training phase (Fig. 2e, left) and showed no significant deficits in the probe trial conducted one week later (Fig. 2e, middle and right). Given that anxiety and depression frequently co-occur, we sought to determine whether *Arhgap39*-deficient mice exhibit depression-like behavior. Accordingly, we performed behavioral assays on another cohort of mice to assess depression-related phenotypes, including the forced swim test, tail suspension test, and sucrose preference test (Fig. 2f). Across all three paradigms, *Arhgap39* cKO mice showed no significant differences compared to WT mice. These results suggest that *Arhgap39* deficits increase anxiety levels without affecting cognitive functions and depression-like behaviors.

### Impaired cognitive functions in aged *Arhgap39* cKO mice

**Fig. 3:**
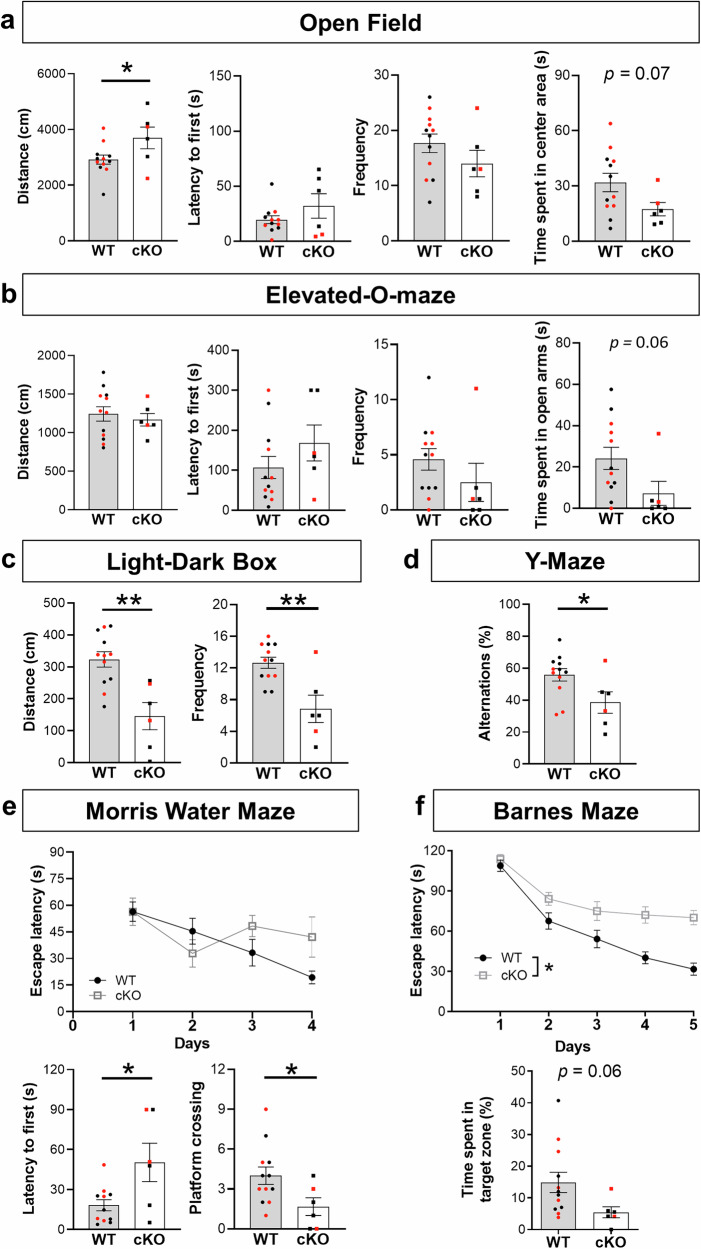
Impaired cognitive functions in aged *Arhgap39* cKO mice. Performance of aged *Arhgap39* cKO mice in **a** Open field test: total distance traveled (t_(16)_ = 2.186, *p* = 0.044), latency to first enter the center (t_(16)_ = 1.36, *p* = 0.192), number of center entries (t_(16)_ = 1.262, *p* = 0.225), and time spent in the center zone (t_(16)_ = 1.907, *p* = 0.074) were analyzed. **b** Elevated-O-maze test: total distance traveled (t_(16)_ = 0.515, *p* = 0.613), latency to first enter the open arms (t_(16)_ = 1.138, *p* = 0.271), number of open arm entries (t_(16)_ = 1.138, *p* = 0.271), and time spent in the open arms (t_(16)_ = 1.953, *p* = 0.068) were analyzed. **c** Light-dark box test: distance traveled in light compartment (t_(16)_ = 3.948, *p* = 0.001) and number of transitions between compartments (t_(16)_ = 3.767, *p* = 0.001) were analyzed. **d** Y-maze: the percentage of spontaneous alternation were analyzed (t_(16)_ = 2.403, *p* = 0.028). **e** Morris Water Maze: the learning curve during the training sessions (mixed-level repeated measures ANOVA, F_(11,176)_ = 1.554, *p* = 0.116), and performance in the probe trial as assessed by number of platform crossings (t_(16)_ = 2.242, *p* = 0.039) and latency to first platform crossing (t_(16)_ = 2.719, *p* = 0.015). **f** Barnes Maze: the learning curve during the training sessions (mixed-level repeated measures ANOVA, F_(4,64)_ = 2.974, *p* = 0.025), and performance in the probe trial as assessed by time spent in target area (t_(16)_ = 1.983, *p* = 0.064). WT 6 males, 6 females; cKO 4 males, 2 females.

In the hippocampus, ARHGAP39 protein levels were the highest in aged brains compared to brains at other ages (Fig. 1c). Adding that increased fur greying was observed in aged *Arhgap39* cKO male, but not female, mice as compared to their littermate controls (male: t_(7)_ = 6.107, *p* < 0.001; female: t_(6)_ = 2.15, *p* = 0.075) (Fig. S5a, b). In the body weight, aged *Arhgap39* cKO mice were even lighter than their WT controls (Fig. S5c). These phenotypes led us to hypothesize that ARHGAP39 might play a critical role in aged mice. To explore this possibility, we thoroughly examined the aged *Arhgap39* cKO mice and their littermate controls. In anxiety tests, aged *Arhgap39* cKO mice displayed a nonsignificant trend toward increased anxiety, showing a tendency to spend less time in the center area of the open field and in the open arms of the elevated-O-maze (Fig. 3a, b). In the light-dark box test, aged cKO mice displayed significantly higher anxiety levels compared to WT mice (Fig. 3c). The increased anxiety-like behavior observed in aged cKO mice is consistent with the phenotypes in younger adults.

Notably, when assessing cognitive abilities, aged *Arhgap39* cKO mice exhibited memory impairments across multiple behavioral assays including the Y-maze, the Morris water maze, and the Barnes maze. In the Y-maze test, aged cKO mice frequently re-entered the same arm, indicating spatial working memory deficits (Fig. 3d). In the Morris water maze, there was no significant difference in overall performance between aged *Arhgap39* cKO and WT mice during the training phase, as analyzed by ANOVA. However, linear regression analysis revealed a significantly flatter learning curve in cKO mice compared to WT controls (test for equality of slopes: F_(1, 68)_ = 4.173, *p* = 0.044), indicating that aged cKO mice exhibited significantly worse spatial learning than WT mice during the training phase. Furthermore, aged cKO mice exhibited impaired spatial memory during the probe trial conducted one week later (Fig. 3e). To rule out the influence of age-related motor deficits, we further assessed cognitive performance using the Barnes maze. During training sessions, aged cKO mice showed reduced spatial learning ability and exhibited lower confidence during the probe trial conducted three days later (Fig. 3f). Together, these results indicate that aged *Arhgap39* cKO mice exhibit significant impairments in both long-term and short-term spatial learning and memory.

Given that aged *Arhgap39* cKO mice exhibited deficits in hippocampal function and synaptic abnormalities, including shortened dendritic length, reduced branching, and increased spine density in neurons [5], we conducted histological analyses to determine whether structural alterations in the hippocampus accompanied these behavioral impairments. NeuN expression in the DG and CA1 regions revealed no significant difference in the thickness or intensity between aged cKO and WT mice, indicating that the overall neuronal architecture remained intact (Fig. S5d). Consistently, MRI analysis showed no evidence of hippocampal atrophy in aged cKO mice (Fig. S5e). Although hippocampal structure was preserved, we detected a significant increase in Iba-1 intensity compared to WT controls, suggesting increased microglial activation, whereas their GFAP expression levels remained comparable to WT mice (Fig. S5f, g).

### Altered stress response and excitatory-inhibitory gene expression in *Arhgap39* cKO mice

Altered plasma cortisol levels and stress-induced dysfunction of the hypothalamic–pituitary–adrenal (HPA) axis are key features of anxiety disorders [28]. Considering that *Arhgap39* cKO mice exhibit elevated anxiety levels and reduced adult hippocampal neurogenesis, we investigated whether ARHGAP39 played a role in regulating the HPA axis reactivity and stress response. To this end, we evaluated several key indicators, including expression of excitatory-, inhibitory-, and glucocorticoid receptor (GR)-related genes, plasma corticosterone levels, and c-Fos expression following a 30-min restraint stress procedure.

**Fig. 4:**
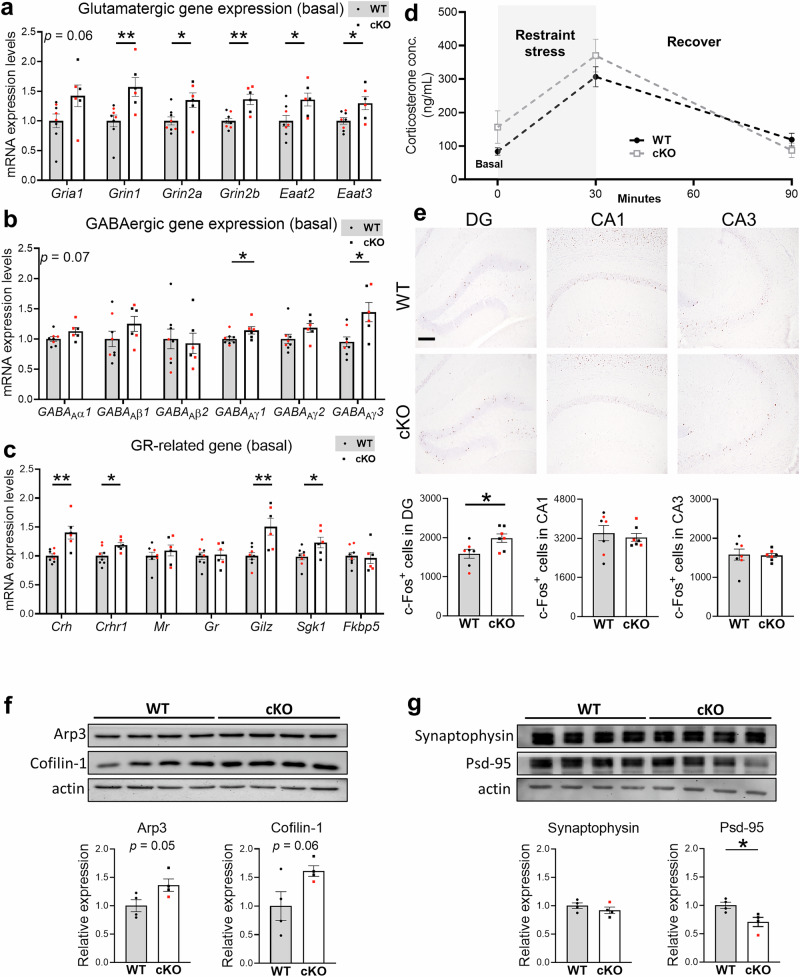
Altered stress response and excitatory-inhibitory gene expression in *Arhgap39* cKO mice. **a** Relative hippocampal mRNA expression levels of glutamatergic-related genes, including *Gria1* (t_(12)_ = 2.055, *p* = 0.062), *Grin1*(U = 3, *p* = 0.004), *Grin2a* (t_(12)_ = 2.637, *p* = 0.021), *Grin2b* (t_(12)_ = 3.998, *p* = 0.001), *Eaat2* (t_(12)_ = 2.546, *p* = 0.025), *Eaat3*, (t_(12)_ = 2.624, *p* = 0.022) under basal conditions. **b** Relative hippocampal mRNA expression levels of GABAergic-related genes, including *GABA*_*A*_*α1* (t_(12)_ = 1.953, *p* = 0.074)*, GABA*_*A*_*β1* (t_(12)_ = 1.364, *p* = 0.197)*, GABA*_*A*_*β2* (t_(12)_ = 1.953, *p* = 0.074)*, GABA*_*A*_*γ1* (t_(12)_ = 2.184, *p* = 0.049)*, GABA*_*A*_*γ2* (U = 11, *p* = 0.107), and *GABA*_*A*_*γ3* (t_(12)_ = 2.973, *p* = 0.011) under basal conditions. **c** Relative hippocampal mRNA expression levels of genes related to glucocorticoid receptors, including *Crh* (t_(12)_ = 3.872, *p* = 0.002), *Crhr1* (t_(12)_ = 2.56, *p* = 0.025), *Mr* (t_(12)_ = 0.848, *p* = 0.412), *Gr* (t_(12)_ = 0.213, *p* = 0.834), *Gilz* (t_(12)_ = 3.562, *p* = 0.003), *Sgk1* (t_(12)_ = 2.453, *p* = 0.03)*, Fkbp5* (t_(12)_ = 0.344, *p* = 0.736) under basal conditions. **a-c** All the data were normalized to the WT group. WT 5 males, 3 females; cKO 3 males, 3 females. **d** Plasma corticosterone levels were measured at three time points: under basal conditions, after 30 min of restraint stress, and following 60 min of recovery (mixed-level repeated measures ANOVA, F_(2,36)_ = 3.035, *p* = 0.06) WT 6 males, 6 females; cKO 4 males, 2 females. **e** Representative images of immunohistochemical staining of c-Fos in the hippocampus under stress conditions. C-Fos expression was examined in the DG (t_(12)_ = 2.551, *p* = 0.025), CA1 (t_(12)_ = 0.494, *p* = 0.63), and CA3 (t_(12)_ = 0.158, *p* = 0.876) subregions. Scale bar = 100 μm. WT 4 males, 3 females; cKO 4 males, 3 females. **f** Western blot analysis of hippocampal lysates, including Arp3 (t_(6)_ = 2.378, *p* = 0.055) and Cofilin-1 (t_(6)_ = 2.271, *p* = 0.063). Quantification of protein levels was normalized to β-actin. WT 4 males; cKO 3 males, 1 female. **g** Western blot analysis of hippocampal lysates, including Synaptophysin (t_(6)_ = 1.076, *p* = 0.323) and Psd-95 (t_(6)_ = 2.954, *p* = 0.025). Protein levels were normalized to β-actin. WT 4 males; cKO 3 males, 1 female.

To investigate the molecular changes, two cohorts of mice were used to assess the expression of glutamatergic-, GABAergic-, and GR-related genes in the hippocampus under both basal and stress conditions. Under basal conditions, *Arhgap39* cKO mice showed increased expression of glutamatergic-receptor (*Gria1, Grin1, Grin2a, Grin2b)* and transporter *(Eaat2*, *Eaat3)* genes, suggesting enhanced excitatory drive at the transcriptional level (Fig. 4a). In parallel, genes associated with inhibitory signaling, such as *GABA*_*A*_*γ1*, and *GABA*_*A*_*γ3* were also upregulated (Fig. 4b). Additionally, *Crh* and *Crhr1* mRNA expression levels were elevated in *Arhgap39* cKO mice, along with increased expression of GR-related genes *Sgk1* and *Gilz* (Fig. 4c), indicating dysregulation of glucocorticoid-responsive genes. Under stress conditions, WT and cKO mice underwent 30 min of restraint stress and were sacrificed 60 min after the restraint stress. *Arhgap39* cKO mice maintained increased expression of glutamatergic-related genes (Fig. S6a), while their expression of GABAergic genes became equivalent to the WT controls (Fig. S6b), further supporting an imbalance in excitatory/inhibitory signaling. Moreover, the expression of *Crh* and *Crhr1* remained elevated compared to WT controls, along with upregulation of GR-related genes, including *Gr*, *Gilz*, and *Fkbp5* (Fig. S6c). These transcriptional changes suggest persistent dysregulation of the GR-related genes and a reduced capacity to regulate stress responses in *Arhgap39* cKO mice.

For plasma corticosterone, we collected samples at three time points: under basal conditions, after 30 min of restraint stress, and following 60 min of recovery. Our results showed no significant difference in the plasma corticosterone levels between the groups, either during the stress or recovery phases, despite a trend toward increased corticosterone levels under basal conditions (Fig. 4d).

We next investigated whether stress led to corresponding changes in neural activity within the hippocampus. c*-*Fos, an immediate-early gene, is commonly used as a marker of neuronal activation following a stimulus [29]. To assess neuronal activity in *Arhgap39* cKO mice, we performed c-Fos immunostaining to identify activated neurons. Our results showed a significant increase in the number of c-Fos-positive cells in the DG of cKO mice, but not in the CA1 or CA3 regions (Fig. 4e). Together, these findings suggest that *Arhgap39* deficiency is accompanied by changes in gene expression associated with excitatory and inhibitory regulation, glucocorticoid response, and hippocampal neuronal activity, which may partially underlie the heightened anxiety-like behavior observed in *Arhgap39* cKO mice.

Moreover, to examine the mechanism under *Arhgap39* ablation, we check the downstream pathway of the Rho GTPase family in actin arrangement-related proteins [30]. There is no significant difference in the Rock1, Dia, Cdk5, and Pak1 pathway (Fig. S7a, b). However, we found an increasing trend of Cofilin-1 and Arp3 in *Arhgap39* cKO compared to WT mice (Fig. 4f), indicating morphological changes of the synapse. This is consistent with the previous result of an increased spine density in neuronal *Arhgap39* KO mice [5]. Synapse stability, which is essential for preserving the long-term functional connectivity of neural circuits, is intrinsically linked to synapse morphology [31]. Thus, we investigate the synapse stability by labeling synaptophysin and PSD-95. We found a significant decrease of PSD-95 but not synaptophysin in *Arhgap39* cKO mice (Fig. 4g), suggesting that morphological changes cause loss of synapse stability.

### Anxiolytic effect of hippocampal *Arhgap39* overexpression in chronic stress mouse model

**Fig. 5:**
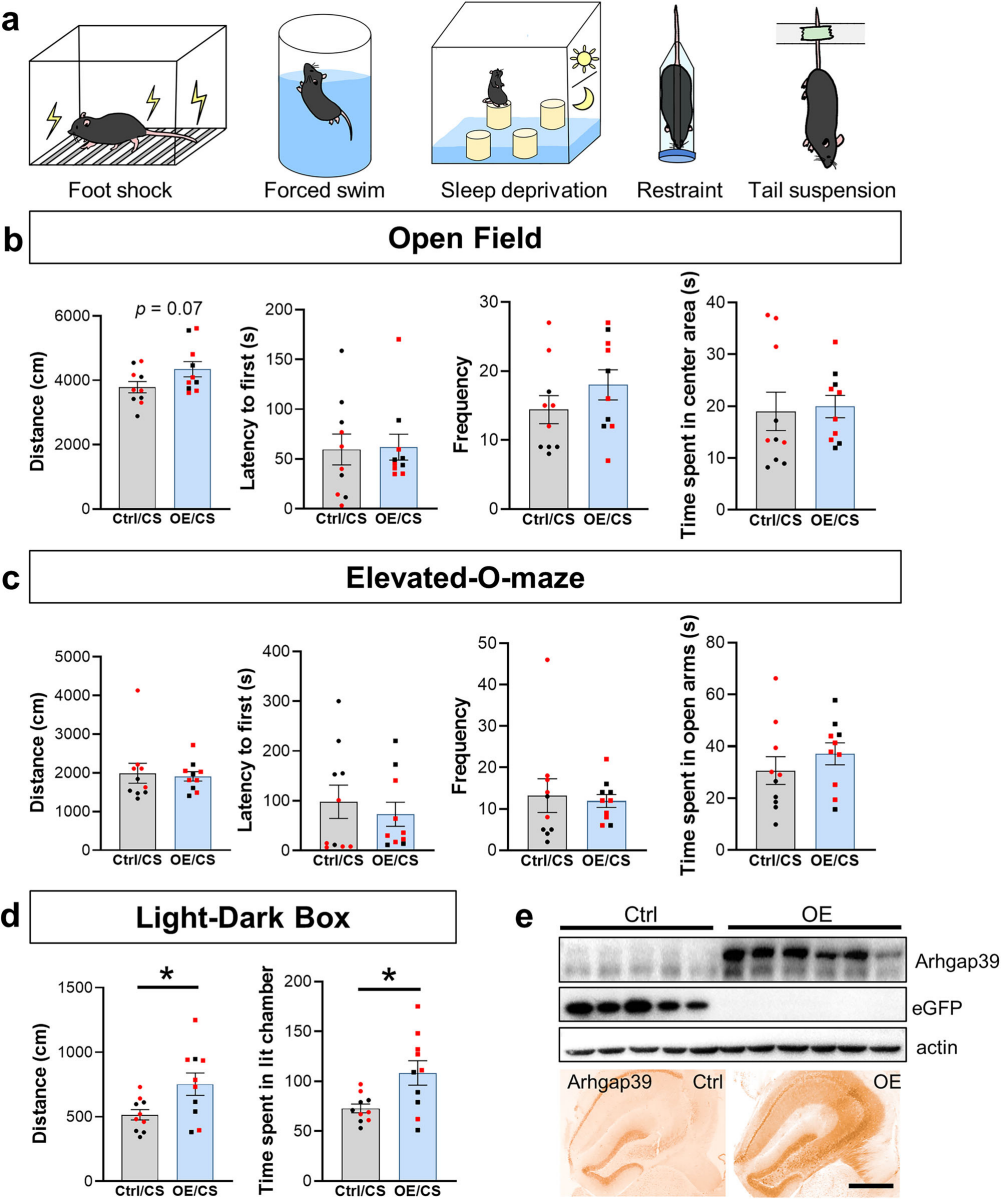
Anxiolytic effect of hippocampal *Arhgap39* overexpression in chronic stress mouse model. Performance of *Arhgap39* overexpressing (OE) mice subjected to chronic stress (CS) **a** Five different stress paradigms used for chronic stress induction, including foot shock, forced swimming, sleep deprivation, restraint stress, and tail suspension. **b** Open Field test: total distance traveled (t_(18)_ = 1.889, *p* = 0.075), latency to first enter the center (U = 44, *p* = 0.684), number of center entries (t_(18)_ = 1.203, *p* = 0.244), and time spent in the center zone (t_(18)_ = 0.216, *p* = 0.83) were analyzed. **c** Elevated-O-maze test: total distance traveled (U = 48, *p* = 0.911), latency to first enter the open arms (t_(18)_ = 0.603, *p* = 0.553), number of open arm entries (U = 43.5, *p* = 0.643), and time spent in the open arms (t_(18)_ = 0.951, *p* = 0.353) were analyzed. **d** Light-dark box test: distance traveled in light compartment (t_(18)_ = 2.463, *p* = 0.024) and time spent in light chamber (t_(18)_ = 2.72, *p* = 0.014) were analyzed. **b–d** Control/CS 5 males, 5 females; OE/CS 4 males, 6 females. (**e**) Hippocampal ARHGAP39 expression level confirmed by Western blot and immunohistochemistry. Representative image shown were obtained from female mouse. Scale bar = 500 μm.

Given that depletion of *Arhgap39* altered the expression of glutamatergic, but not GABAergic, genes under stress conditions, we overexpressed *Arhgap39* specifically in hippocampal excitatory neurons of C57BL/6 mice using an AAV vector driven by the *Camk2a* promoter to further investigate the role of excitatory neuronal *Arhgap39* in anxiety-related behavior. Behavioral tests were conducted one month after the surgery. In all three anxiety-related behavioral tests, *Arhgap39* overexpressing mice did not exhibit any abnormality compared to control mice (Fig. S8a–c). One possible explanation is that the C57BL/6 strain, known to be one of the least anxious inbred mouse strains [32], may mask the potential anxiolytic effects under basal conditions. To overcome this limitation, we employed an unpredictable chronic stress model, a well-established paradigm for inducing stress-related phenotypes. To address this, we subjected all mice to unpredictable chronic stress for one month (Fig. 5a). We found that *Arhgap39* overexpressing mice exhibited less anxiety-like behavior in the light-dark box test, but not the open field test and elevated-O-maze, compared to control mice (Fig. 5b-d). This finding suggests that hippocampal overexpression of *Arhgap39* had an anxiolytic effect in some of the anxiety tests, after chronic stress. The efficiency of *Arhgap39* overexpression was confirmed by Western blot and Immunohistochemical staining (Fig. 5e).

## Discussion

In this study, we investigated the role of hippocampal *Arhgap39* in regulating anxiety-like behavior, stress-related molecular responses, and downstream pathways associated with synaptic organization. *Arhgap39* deficiency in the brain disrupted early-stage of hippocampal development and impaired adult hippocampal neurogenesis. At the behavioral level, adult *Arhgap39* cKO mice exhibited increased anxiety-like behavior without impairments in learning. While in aged cKO mice, deficits in spatial learning and memory occurred. At the molecular level, *Arhgap39* cKO mice showed altered glutamatergic- and GABAergic-related gene expression. Our results reveal that *Arhgap39* deficiency alters actin-regulating proteins and reduces PSD-95 levels, indicating impaired synaptic stability. Moreover, overexpression of *Arhgap39* in hippocampal neurons reduced anxiety-like behavior in the light-dark box test in mice exposed to chronic stress, suggesting Ar*hgap39* as a potential therapeutic target for anxiety-related disorders.

Since our cKO mice were generated using Sox1 promoter-driven Cre recombinase, Cre activity is present during early neural tube development, resulting in the complete knockout of *Arhgap39* throughout the brain. This allowed us to explore the role of *Arhgap39* in early postnatal neurodevelopment. Although both the cKO mice and their littermate controls were homozygous for the floxed allele, we cannot completely exclude the possibility that the floxed allele may exert hypomorphic effects compared with the non-floxed allele. In this study, we observed that *Arhgap39* cKO mice exhibited an increased number of ectopic Sox2-, Pax6-, and Ki67- positive cells in the DG hilus during early development, indicating the presence of ectopic neuronal progenitor cells. Yet the numbers of Tbr2- and Prox1-positive cells were similar to those in WT mice. These findings imply that the absence of *Arhgap39* may disrupt the progression of neuronal maturation. This developmental progression is similar to previous findings in AP2γ-deficient mice, where constitutive deletion of the gene impaired hippocampal neurogenesis and disrupted limbic-cortical connectivity, leading to anxiety-like behavior and memory deficits beginning in the juvenile stage and continuing into adulthood [33].

Genetic deletion of *Arhgap39* in neurons led to profound synaptic abnormalities accompanied by impaired LTP in hippocampal slices, reflecting substantial disruption of synaptic strength and plasticity [5]. The convergence of structural and functional impairments highlights the pivotal role of *Arhgap39* as a molecular regulator that integrates dendritic architecture with synaptic adaptability. In this study, we further found that a significant reduction in hippocampal neurogenesis in *Arhgap39* cKO mice. Adult hippocampal neurogenesis has long been associated with both learning and anxiety-like behaviors [34, 35], despite that whether the reduced level of adult hippocampal neurogenesis is directly linked to increased anxiety or impaired learning remains a subject of ongoing debate. In our study, we found that the expression of ARHGAP39 in the hippocampus progressively increased with age, suggesting its importance not only during development but also throughout the aging process. This age-related expression pattern may partially explain why *Arhgap39* cKO mice showed no significant learning impairments in young adulthood but exhibited impaired performance in spatial learning tasks as they aged.

*Arhgap39* cKO mice exhibited elevated hippocampal *Crh* and *Crhr1* mRNA expression, along with a significant increase in c-Fos-positive neurons in the DG following stress exposure. As c-Fos is a well-established immediate early gene and a marker of neuronal activation, these findings indicate enhanced hippocampal neuronal responsiveness to stress in the absence of *Arhgap39* [36]. Hyperactivation of the CRH-CRHR1 signaling pathway has been shown to disrupt stress-related regulatory mechanisms and enhance neuronal excitability within the hippocampus [37–40]. Chronic overactivation of CRHR1 signaling has been associated with synaptic loss and dendritic remodeling, which may compromise neuronal connectivity and circuit stability [41]. Supporting this notion, previous study reported reduced dendritic complexity and altered spine morphology in *Arhgap39*-deficient neurons [5]. Together, these findings suggest that dysregulated hippocampal CRH-CRHR1 signaling may contribute to synaptic abnormalities and heightened anxiety-like behaviors observed in *Arhgap39*-deficient mice.

Despite unchanged circulating corticosterone levels, alterations in hippocampal *Crh, Crhr1*, and glucocorticoid receptor–related gene expression suggest that stress-related dysregulation in *Arhgap39* cKO mice occurs primarily at the level of central glucocorticoid responsiveness rather than systemic HPA axis output. While PVN-mediated endocrine control of corticosterone release appears largely preserved, hippocampal circuits may exhibit altered sensitivity or transcriptional responses to equivalent glucocorticoid exposure. Such central–peripheral dissociation provides a framework for understanding how *Arhgap39* deficiency selectively impacts hippocampal stress-related signaling, thereby predisposing hippocampal circuits to downstream structural and functional alterations.

In addition to *Crh* and *Crhr1*, we further analyzed the mRNA expression level of several GR-related genes that are highly expressed in the hippocampus under both stress and non-stressed conditions. Among these, glucocorticoid receptor (GR, gene name: *Nr3c1)* and mineralocorticoid receptor (MR, gene name: *Nr3c2)* are two major receptors mediating glucocorticoid signaling in the brain [42]. Particularly, MR has a higher binding affinity for glucocorticoids compared to GR and is largely occupied even under basal corticosterone levels [43, 44]. For this reason, MR is believed to regulate basal glucocorticoid signaling and is not further induced during stress. In contrast, GR exhibits a lower affinity and is primarily activated during stress when circulating corticosterone level rises significantly. GR is essential for mediating the negative feedback regulation of the HPA axis and facilitating post-stress recovery [43, 45, 46]. In our study, we found that hippocampal *NR3c*1/GR mRNA expression was significantly upregulated under stress, while Nr3c2/MR expression remained unchanged, indicating the dysregulation of negative feedback in *Arhgap39* cKO mice. However, we acknowledge that the current data do not directly address how ARHGAP39 modulates the activity or connectivity of the relevant neural circuits. Incorporating approaches such as in vivo electrophysiology, optogenetics, chemogenetics, or tract tracing will be essential for further elucidating the underlying mechanisms.

Several GABAergic genes were upregulated under basal conditions but returned to WT levels following stress exposure. The biological significance of this state-dependent change remains unclear. Based on the current data, we cannot determine whether these differences reflect compensatory homeostatic responses, altered inhibitory plasticity, or context-dependent regulation related to stress. Further studies will be required to clarify the functional relevance of this observation.

In this study, we found a significant increase in GFAP intensity in several hippocampal subregions in adult *Arhgap39* cKO mice, indicating astrocyte activation. This activation likely reflects a compensatory response to excessive glutamatergic transmission and synaptic dysfunction. Astrocytes play a central role in maintaining synaptic homeostasis by clearing extracellular glutamate through excitatory amino acid transporters (EAATs), thereby preventing excitotoxicity and maintaining synaptic stability [47, 48]. Consistent with this function, our results showed alterations in *Gria1*, *Grin1*, *Grin2a*, *Grin2b*, *Eaat2*, and *Eaat3* expression in *Arhgap39* cKO mice. These molecular changes suggest that astrocytic hyperactivation is a consequence of glutamate overproduction, which may in turn lead to impaired synaptic stability, as supported by elevated levels of Arp3 and Cofilin-1 observed in our model.

Past studies have implicated hippocampal astrocytes in the regulation of anxiety-related behavior in a bidirectional manner [22, 25]. Astrocytic activation in the hippocampus increases when mice are exposed to stressful environments, such as the open arms of the elevated plus maze, whereas activation decreases in safer environments like the closed arms. Moreover, experimental manipulation of astrocyte activity has been shown to modulate anxiety-like behavior [22]. On the other hand, Iba1 expression remained unchanged in most hippocampal areas, except for a mild increase specifically in the CA3 region. The hypophagocytic state of microglia has been observed in mice with higher anxiety-like behavior [23], which may partially explain the unchanged microglia intensity in our study. These results suggest that the observed increase in GFAP and the unchanged Iba1 intensity are unlikely to result from a neuroinflammatory response. Instead, it may reflect astrocyte-specific regulatory mechanisms, potentially involving disrupted tripartite synapse function. Supporting this possibility, we found a marked enhancement of glutamatergic transmission in *Arhgap39* cKO mice compared to WT controls. This hyperexcitability may result from impaired astrocytic regulation of synaptic activity, highlighting the critical role of astrocytes in maintaining excitatory/inhibitory balance in the hippocampus. Such dysregulation could contribute to the heightened anxiety-like behavior observed in our *Arhgap39* cKO mouse model.

To evaluate the therapeutic potential of ARHGAP39, we built on the finding that *Arhgap39* depletion alters glutamatergic-related genes, but not GABAergic genes, under stress conditions. Based on this, we overexpressed *Arhgap39* specifically in hippocampal excitatory neurons. Following chronic stress exposure, mice overexpressing *Arhgap39* displayed reduced anxiety-like behavior in the light-dark box test, but not in the open field or elevated O-maze tests. The absence of a detectable phenotype in the open field and elevated O-maze tests may result from the stress induced by surgery and chronic stress, suggesting that the procedure itself could obscure behavioral alterations observable under less stressful conditions. In addition, our study showed that *Arhgap39* depletion affected the activation of astrocytes and microglia in the hippocampus under baseline condition. For future studies, increasing *Arhgap39* expression across multiple cell types may offer a promising avenue for developing treatments that go beyond anxiety and depressive disorders.

Taken together, our study identifies *Arhgap39* as a key regulator of hippocampal development, synaptic stability, and stress reactivity. *Arhgap39* deficiency leads to impaired neuronal development, disrupted synaptic organization, and heightened anxiety-like behavior in mice. In addition, hippocampal overexpression of *Arhgap39* alleviates chronic stress-induced anxiety, as measured by the light-dark box test, further supporting its potential therapeutic relevance.

## Supplementary information

Supplementary information

## Data Availability

Data is provided within manuscript or supplementary files.
