# Supplementary material for: ARHGAP39 plays an essential role in anxiety-like behavior and stress response
Source: Transl Psychiatry. 2026 May 9;16:321. doi: 10.1038/s41398-026-04088-1 (PMC13323729; doi:10.1038/s41398-026-04088-1)
Supplement: Supplementary file 1 — Supplementary information [file 41398_2026_4088_MOESM1_ESM.docx]

**Supplementary information**

**Title: ARHGAP39 Plays an Essential Role in Anxiety-Like Behavior and Stress Response**

Shin-Meng Deng, Yu-Ju Chen, Wei-Chien Hung, Kuan-Yu Wu, Wei-Che Chang, Irene Han-Juo Cheng, Mau-Sun Chang, Guo-Jen Huang

Contents

Supplementary Methods and Materials ---------------------------------------------------------1-3

Figures S1-S8 -------------------------------------------------------------------------------------4-16

**Supplementary Methods and Materials**

**Chronic stress paradigm**

To induce chronic stress, mice were subjected to five different stressors: foot shock, forced swim, restraint stress, tail suspension, and sleep deprivation. One stressor was applied per day, and the type of stress was randomly selected each day. Each stressor was administered once per week, ensuring that all five stress types were completed within each weekly cycle. This protocol was repeated for four consecutive weeks.

*Foot shock*

Mice were placed in a shock chamber equipped with a metal grid floor (Med Associates). Each session consisted of three foot-shocks (0.5 mA, 30 seconds per shock), with variable inter-shock intervals ranging from 1 to 10 minutes. The timing of each shock was randomized to enhance unpredictability.

*Sleep deprivation*

Mice were kept awake for at least 8 hours during the light phase. Animals were placed in an acrylic box containing multiple small cylindrical platforms (diameter = 3.5 cm, height = 5 cm). The floor of the box was filled with water to a depth of at least 2 cm, such that each mouse had to remain standing on a platform to avoid contact with water. The limited surface area of each platform prevented the mice from lying down or entering a restful posture. Throughout the procedure, animals were continuously monitored. If a mouse fell into the water, it was immediately placed back onto a dry platform.

*Restraint stress*

Mice were placed in well-ventilated plastic restrainers that restricted body movement without causing pain. Each restraint session lasted for 4-6 hours. Mice were closely monitored during the procedure and returned to their home cages immediately afterward.

**Novel Object Recognition**

Novel object recognition (NOR) was performed in a square chamber (30 × 30 cm) made of plastic walls. During the habituation phase, mice were allowed to freely explore the empty chamber for 10 minutes. On the next day, during the familiarization phase, two identical objects were placed in the chamber, and each mouse was allowed to explore for 10 minutes. After a retention interval of 24 hours, one of the familiar objects was replaced with a novel object of similar size but different shape and texture. Mice were allowed to explore the chamber for 10 minutes. Exploration was defined as directing the nose toward the object at a close distance or touching the object with the nose. Sitting on or climbing on the object was not considered exploration. Recognition memory performance was expressed as the discrimination index (DI), calculated as:

*D.I. = (time exploring novel object − time exploring familiar object) / total exploration time.*

**Contextual Fear Conditioning**

Contextual fear conditioning was conducted in a conditioning chamber equipped with a stainless-steel grid floor connected to a shock generator. During the training session, each mouse was placed in the chamber and allowed to explore freely for 3 minutes before receiving a footshock (0.3 mA, 3 seconds). After the shock, mice remained in the chamber for an additional 30 seconds before being returned to their home cage. Twenty-four hours later, mice were returned to the same chamber for 5 minutes without a footshock to assess contextual fear memory. Freezing behavior, defined as the absence of all movement except respiration, was recorded throughout the session, and the percentage of freezing time was used as an index of contextual fear memory.

**Immunohistochemistry**

Brain tissues were fixed in 4% paraformaldehyde at 4°C overnight, and dehydrated in 25% sucrose prepared in phosphate-buffered saline (PBS). Coronal brain sections (40 µm thick) were mounted onto SuperFrost Plus slides (Thermo Fisher, 630-0950). Slides were air-dried overnight at room temperature prior to antigen retrieval, which was performed by incubating the slides in 0.01 M citrate buffer (pH 6.0) at 95 °C for 20-40 minutes. Endogenous peroxidase activity was quenched with 3% hydrogen peroxide (Honeywell Fluka, 31642) for 10 minutes. After rinsing with PBS, sections were incubated 4-6 hours at room temperature with the following primary antibodies: DCX, 1:300, sc-8066, Santa Cruz; Ki67, 1:1500, ab16667, Abcam; SOX2, 1:300, ab97959, Abcam; PAX6, 1:1000, ab2237, Sigma-Aldrich; TBR2, 1:1000, ab23345, Abcam; Prox1, 1:1000 AB5475, Sigma-Aldrich; c-Fos, 1:1500, sc-8047, Santa Cruz. Signal detection was performed using the avidin-biotin complex (ABC) method (Vector Laboratories) and visualized with 3,3’-diaminobenzidine (DAB, Sigma-Aldrich). Sections were counterstained with hematoxylin (Scytek), dehydrated, and coverslipped.

For the immunofluorescent staining, sections were incubated 4-6 hours at room temperature with the following primary antibodies: Iba1, 1:500, 019-19741, Wako; GFAP, 1:1000, G3893, Sigma; NeuN, 1:1000, MAB377, Sigma-Aldrich; BrdU (Bromodeoxyuridine), 1:1500, ab6326, Abcam. CTIP2, 1:1000, ab18456, Abcam. PV, 1:200, MAB1572, Sigma-Aldrich. For NeuN/BrdU double staining, DNA denaturation was performed by incubating with 2 N HCl at 37 °C for 15 minutes, followed by neutralization with 0.1M boric acid (pH = 8.5, Honeywell Fluka,31457) for 5 minutes at room temperature. After washing with PBS, sections were incubated with the fluorescent secondary antibody (Alexa Fluor 488: A11008, Invitrogen; Alexa Fluor 594, A11032, Invitrogen; Alexa Fluor 568, A11011, Invitrogen) for 1 hour at room temperature. Finally, slides were coverslipped with Fluoromount-G (100-20, SouthernBiotech, AL, USA).

**Supplementary Figures**


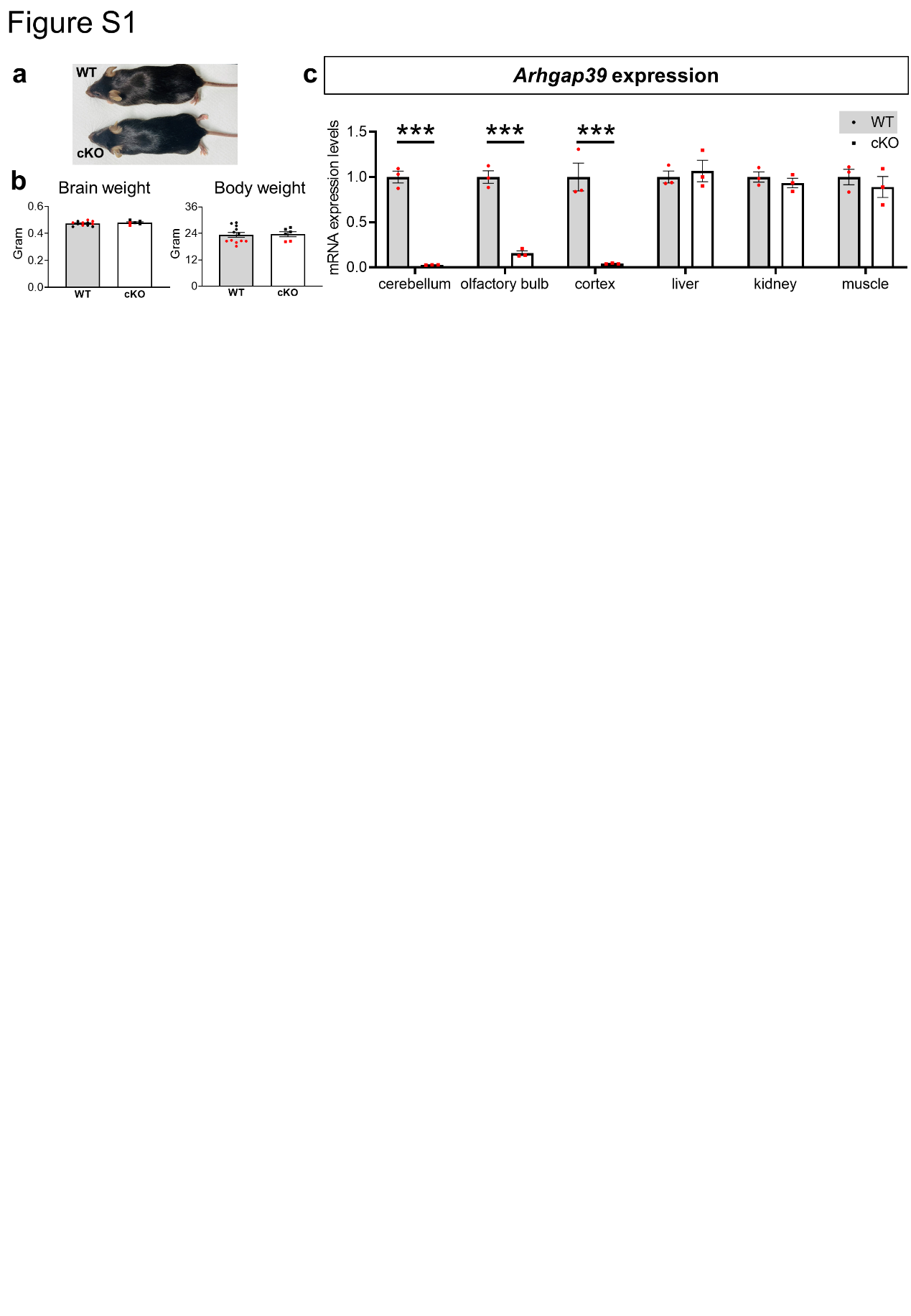


**Fig. S1. Comparison of body and brain weight and regional *Arhgap39* mRNA expression in WT and cKO mice.**

(**a**) Representative images of WT and *Arhgap39* cKO mice. (**b**) Quantification of brain weight (t_(16)_ = 0.857, *p* = 0.403) and body weight (t_(16)_ = 0.184, *p* = 0.856) in WT and *Arhgap39* cKO mice. WT 6 males, 6 females; cKO 4 males, 2 females. (**c**) *Arhgap39* mRNA expression in different brain regions and peripheral tissues. Expression levels in each group were normalized to their respective control groups. Cerebellum: t_(4)_ = 14.95, *p* < 0.001, Olfactory bulb: t_(4)_ = 11.4, *p* < 0.001, Cortex: t_(4)_ = 6.226, *p* = 0.003, Liver: t_(4)_ = 0.491, *p* = 0.649, Kidney: t_(4)_ =0.846, *p* = 0.445, Muscle: t_(4)_ = 0.766, *p* = 0.486, N= 3 females per group.


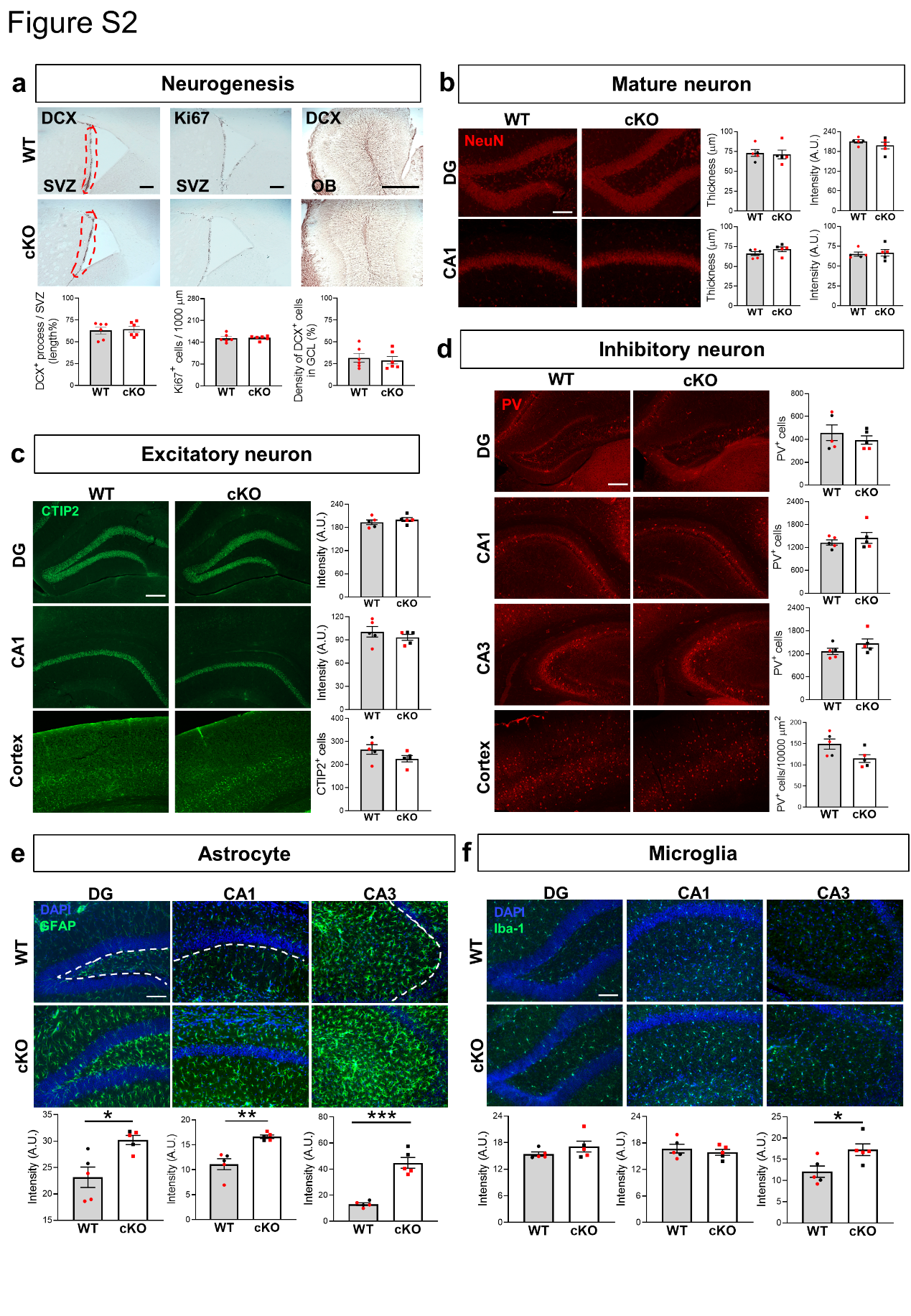


**Fig. S2. Neurogenesis, excitatory–inhibitory markers, and glial changes of *Arhgap39* cKO mice.**

(**a**) Quantification of markers of neurogenesis in SVZ (DCX: t_(10)_ = 0.291, *p* = 0.776; Ki67: t_(10)_ = 0.209, *p* = 0.838) and olfactory bulb (t_(10)_ = 0.435, *p* = 0.672). N = 6 females. Scale bar = 200 μm. (**b**) Immunofluorescence staining of NeuN in the DG and CA1 regions; thickness (DG: t_(8)_ = 0.255, *p* = 0.805; CA1: t_(8)_ = 1.413, *p* = 0.195) and fluorescence intensity of NeuN-positive cell layers (DG: t_(8)_ = 1.152, *p* = 0.282; CA1: t_(8)_ = 0.255) were analyzed. WT 2 males, 3 females; cKO 2 males, 3 females. Scale bar = 100 μm. (**c**) Quantification of CTIP2 fluorescence intensity in the DG (t_(8)_ = 0.856, *p* = 0.416), CA1 (t_(8)_ = 0.922, *p* = 0.385), and cortex (t_(8)_ = 1.6, *p* = 0.148). WT 2 males, 3 females; cKO 3 males, 2 females. Scale bar = 200 μm. (**d**) Quantification of PV-positive cells in the DG (t_(8)_ = 0.819, *p* = 0.436), CA1 (t_(8)_ = 0.756, *p* = 0.471), CA3 (t_(8)_ = 1.426, *p* = 0.191), and cortex (t_(8)_ = 2.267, *p* = 0.053). WT 2 males, 3 females; cKO 3 males, 2 females. Scale bar = 100 μm. (**e**) Quantification of GFAP fluorescence intensity in the DG (t_(16)_ = 0.393, *p* = 0.699), CA1 (t_(16)_ = 0.446, *p* = 0.661), and CA3 regions (t_(16)_ = 0.516, *p* = 0.612), restricted to the inner side of the cell layer. Region boundaries are indicated by white dashed lines. (**f**) Quantification of Iba1 fluorescence intensity in the DG (t_(16)_ = 4.417, *p* < 0.001), CA1(t_(16)_ = 2.983, *p* = 0.008), and CA3 (t_(16)_ = 1.236, *p* = 0.234) regions. (**e**)-(**f**) WT 2 males, 3 females; cKO 2 males, 3 females. Scale bar = 100 μm. A.U. intensity arbitrary unit.

**
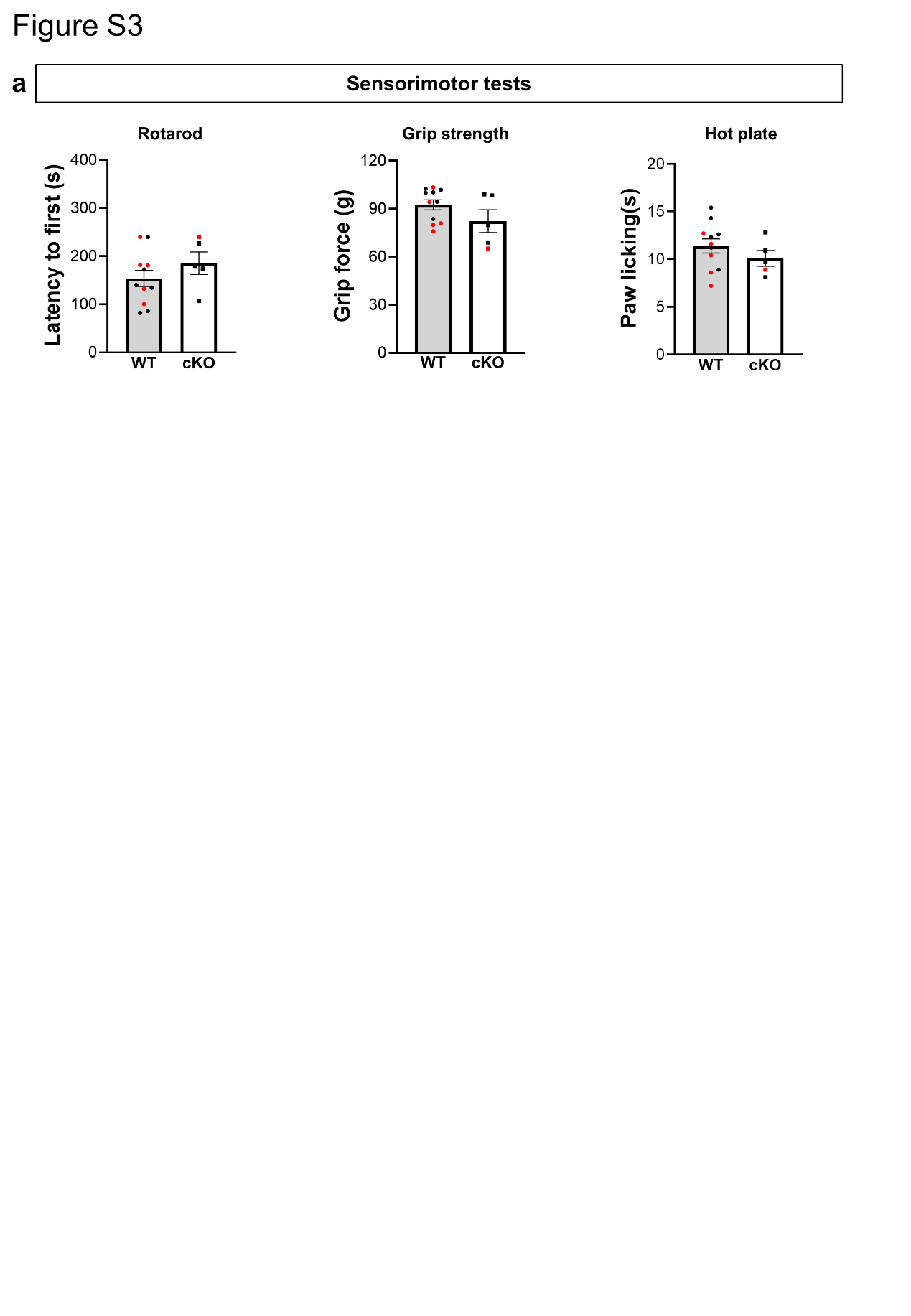
**

**Fig. S3. Sensorimotor behaviors of adult *Arhgap39* cKO mice**

**(a**) Sensorimotor tests of *Arhgap39* cKO mice, including Rotarod (t_(14)_ = 1.085, *p* = 0.296), Grip strength (t_(14)_ = 1.544, *p* = 0.145), and Hot plate test (t_(14)_ = 1.045, *p* = 0.313). WT 6 males, 5 females; cKO 4 males, 1 female.

**
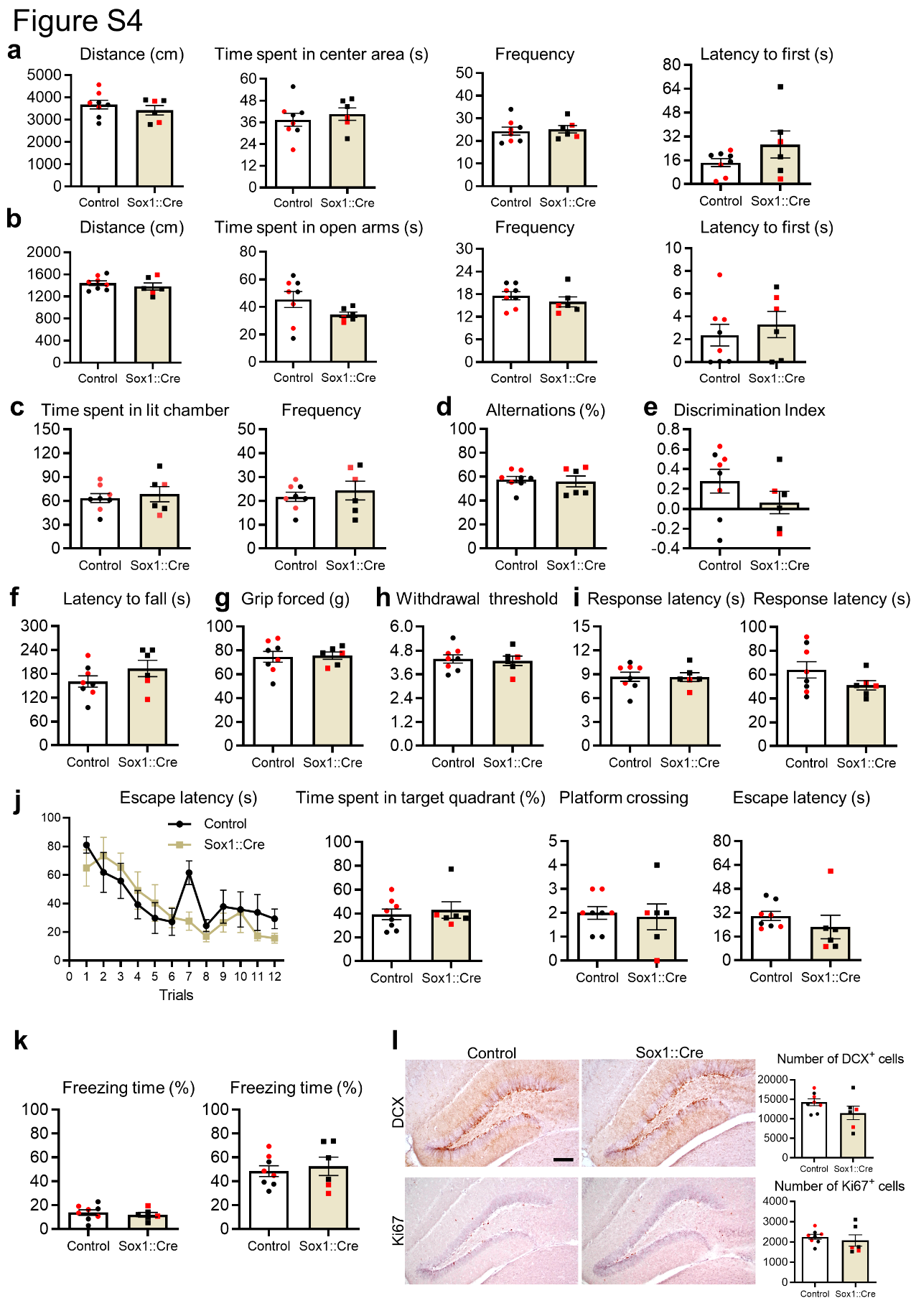
**

**Fig. S4. Behavioral and neurogenesis analyses of *Sox1::Cre* mice compared with littermate control mice**

C57BL/6 mice were crossed with *Sox1::Cre* mice to generate *Sox1::Cre* transgenic mice and *Cre*-negative littermate controls (C57BL/6 background). This cohort was subsequently used for the following experiments. (**a**) Open Field test: total distance traveled (t_(12)_ = 0.89, *p* = 0.39), time spent in the center zone (t_(12)_ = 0.613, *p* = 0.55), number of center entries (t_(12)_ = 0.314, *p* = 0.758), and latency to first center entry (t_(12)_ = 1.452, *p* = 0.172) were analyzed. (**b**) Elevated-O-maze test: total distance traveled (t_(12)_ = 0.816, *p* = 0.43), time spent in the open arms (t_(12)_ = 1.579, *p* = 0.14), number of open arm entries (t_(12)_ = 0.976, *p* = 0.348), and latency to first open arm entry (t_(12)_ = 0.624, *p* = 0.544) were analyzed. (**c**) Light-dark box test: time spent in lit compartment (t_(12)_ = 0.467, *p* = 0.648) and number of transitions between compartments (t_(12)_ = 0.636, *p* = 0.536) were analyzed. (**d**) Y-maze: percentage of spontaneous alternation were analyzed (t_(12)_= 0.322, *p* = 0.752). (**e**) Novel Object Recognition test: discrimination index were analyzed (t_(12)_ = 1.28, *p* = 0.224). (**f–i**) Sensorimotor tests (**f**) Rotarod (t_(12)_ = 1.362, *p* = 0.198), (**g**) Grip strength (t_(12)_ = 0.175, *p* = 0.867), (**h**) von Frey test (t_(12)_ = 0.288, *p* = 0.777), and (**i**) Hot plate test: latency to paw licking (t_(12)_ = 0.081, *p* = 0.936) and latency to jumping (t_(12)_ = 0.155, *p* = 0.163). (**j**) Morris Water Maze: the learning curve during the training sessions (mixed-level repeated measures ANOVA, F_(11,132)_ = 1.062, *p* = 0.397) and the performance in the probe trial as assessed by time spent in the target quadrant (t_(12)_ = 0.464, *p* = 0.651), number of platform crossings (t_(12)_ = 0.298, *p* = 0.77), and latency to first platform crossing (t_(12)_ = 0.971, *p* = 0.35). (**k**) Contextual fear conditioning: freezing percentage before conditioning (left, t_(12)_ = 0.595, *p* = 0.562) and after conditioning (right, t_(12)_ = 0.474, *p* = 0.643) were analyzed. (**l**) Staining of adult neurogenesis markers in the dentate gyrus. Markers examined include DCX (t_(12)_ = 1.547, *p* = 0.147) and Ki67( t_(12)_ = 0.657, *p* = 0.523). Scale bar = 200 μm. Littermate controls: 4 males, 4 females; *Sox1::Cre* 4 males, 2 females.


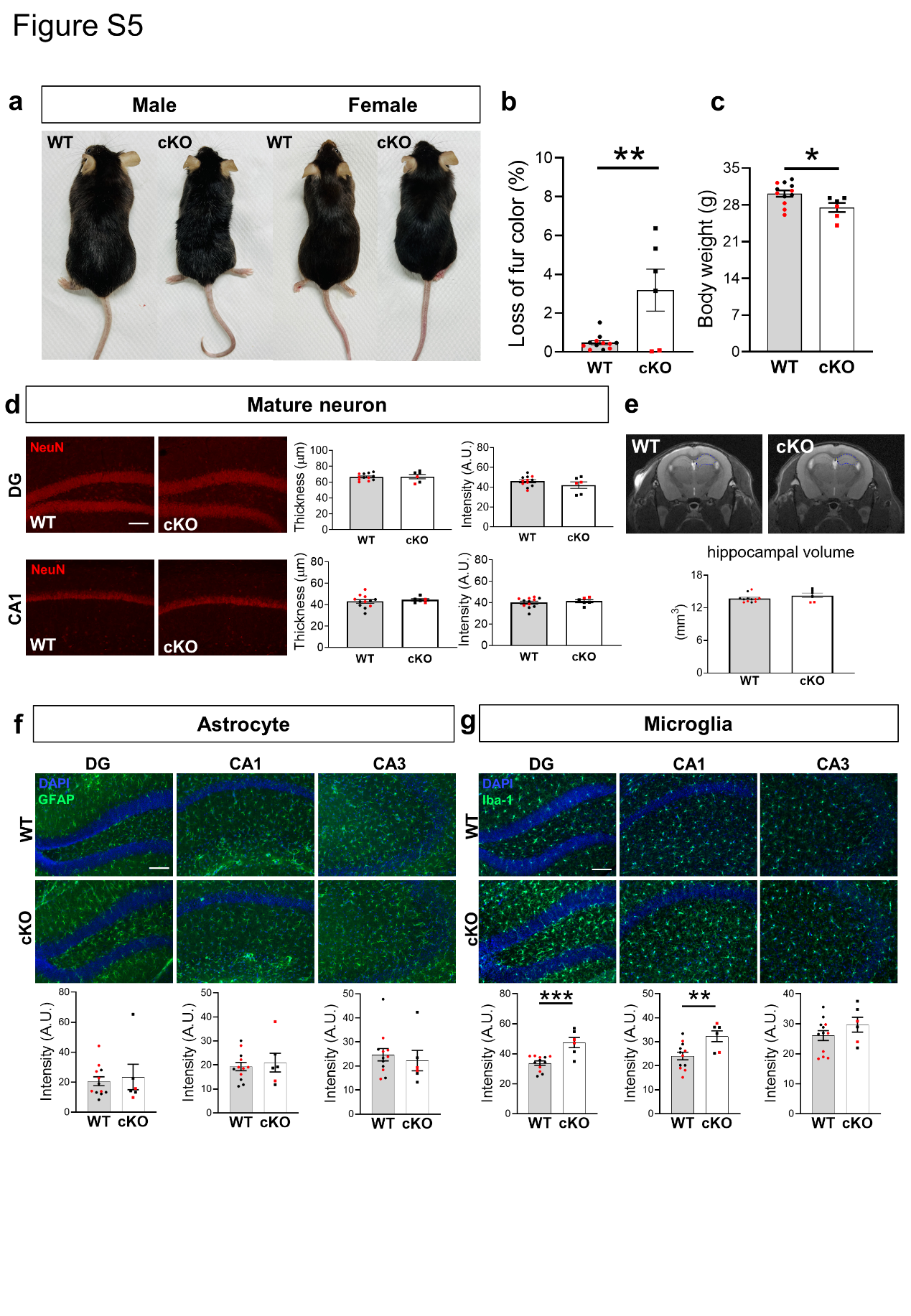


**Fig. S5. Morphological and cellular characterization of aged *Arhgap39* cKO mice**

(**a**) Representative images of aged *Arhgap39* cKO and WT mice. (**b**) Percentage of fur color loss on the back of aged *Arhgap39* cKO mice compared to WT mice. t_(16)_ = 3.572, *p* = 0.002 (**c**) Quantification of body weight in aged *Arhgap39* cKO and WT mice. (**d**) Immunofluorescence staining of NeuN in the DG and CA1 regions; thickness (DG: t_(16)_ = 0.148, *p* = 0.883; CA1: t_(16)_ = 0.535, *p* = 0.599) and fluorescence intensity of NeuN-positive cell layers (DG: t_(16)_ = 1.244, *p* = 0.231; CA1: t_(16)_ = 0.63, *p* = 0.537) were analyzed. (**e**) MRI of whole brains from aged *Arhgap39* cKO and WT mice. The volume of hippocampus was analyzed (t_(16)_ = 1.242, *p* = 0.232) . (**f**) Quantification of GFAP fluorescence intensity in the DG (t_(16)_ = 0.393, *p* = 0.699), CA1 (t_(16)_ = 0.446, *p* = 0.661), and CA3 (t_(16)_ = 0.516, *p* = 0.612) regions, restricted to the inner side of the cell layer. (**g**) Quantification of Iba1 fluorescence intensity in the DG (t_(16)_ = 4.417, *p* < 0.001), CA1 (t_(16)_ = 2.983, *p* = 0.008), and CA3 (t_(16)_ = 1.236, *p* = 0.234) regions, restricted to the inner side of the cell layer. WT 6 males, 6 females; cKO 4 males, 2 females. Scale bar = 100 μm. A.U. intensity arbitrary unit.


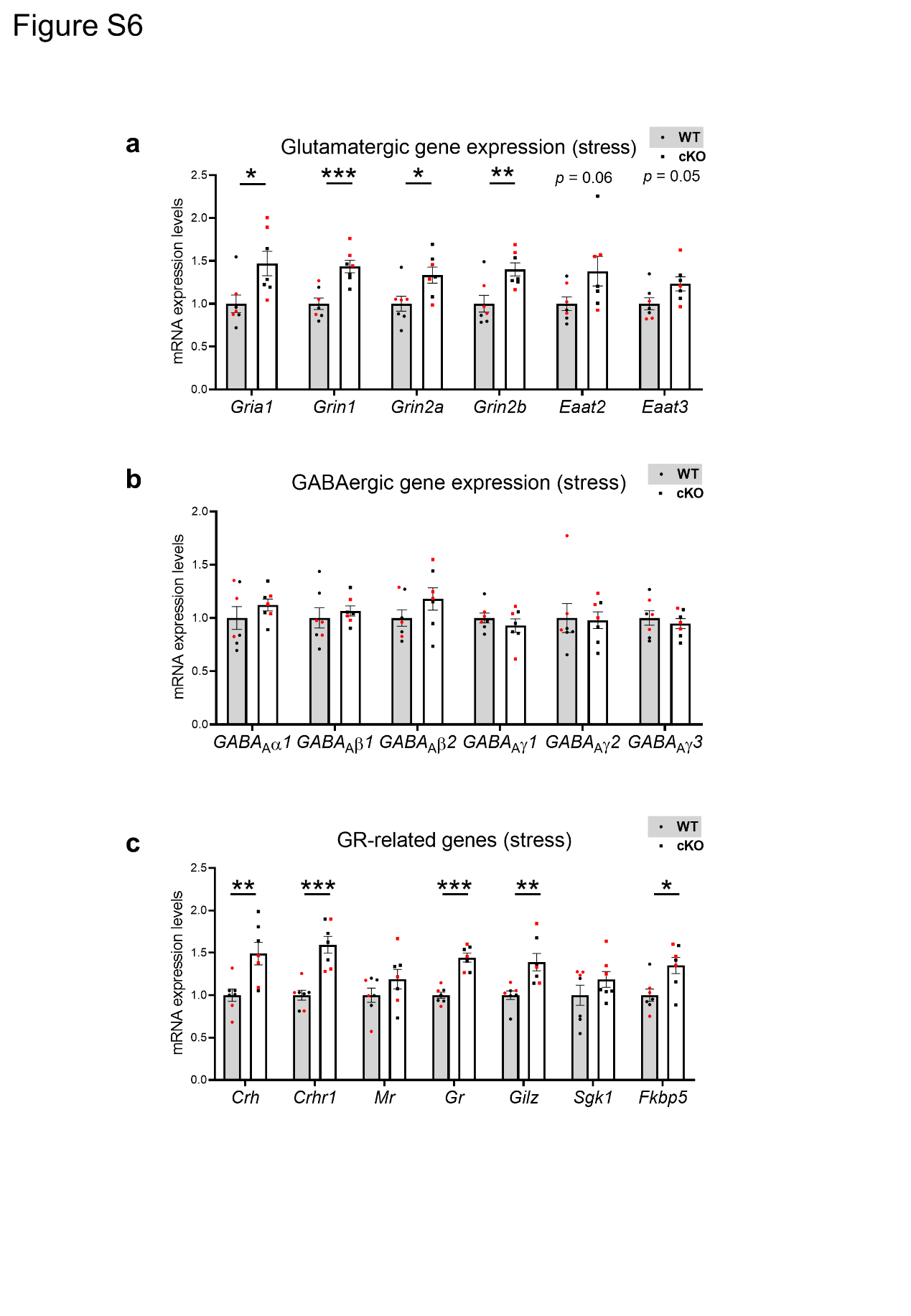


**Fig. S6. Altered stress response and excitatory-inhibitory signaling in *Arhgap39* cKO mice under stress conditions.**

(**a**) Analysis of glutamatergic–related gene expression in the hippocampus of *Arhgap39* cKO mice, including *Gria1* (t_(12)_ = 2.685, *p* = 0.019), *Grin1*(t_(12)_ = 4.404, *p* < 0.001), *Grin2a* (t_(12)_ = 2.611, *p* = 0.022), *Grin2b* (t_(12)_ = 3.218, *p* = 0.007), *Eaat2* (t_(12)_ = 1.995, *p* = 0.069), *Eaat3,* (t_(12)_ = 2.159, *p* = 0.051) under stress conditions. (**b**) Analysis of GABAergic–related gene expression in the hippocampus of *Arhgap39* cKO mice including *GABA_A_α1* (t_(12)_ = 1.019, *p* = 0.328)*, GABA_A_β1*(t_(12)_ = 0.616, *p* = 0.548)*, GABA_A_β2* (t_(12)_ = 1.372, *p* = 0.195)*, GABA_A_γ1* (t_(12)_ = 0.916, *p* = 0.377)*, GABA_A_γ2* (t_(12)_ = 0.128, *p* = 0.9)*,* and *GABA_A_γ3* (t_(12)_ = 0.643, *p* = 0.532)*,* under stress conditions. (**c**) Analysis of GR–related gene expression in the hippocampus of *Arhgap39* cKO mice, including *Crh* (t_(12)_ = 3.25, *p* = 0.007), *Crhr1* (t_(12)_ = 5.179, *p* < 0.001), *Mr* (t_(12)_ = 1.322, *p* = 0.21), *Gr* (t_(12)_ = 7, *p* < 0.001), *Gilz* (t_(12)_ = 3.397, *p* = 0.005), *Sgk1* (t_(12)_ = 1.228, *p* = 0.243)*, Fkbp5* (t_(12)_ = 2.906, *p* = 0.013) under stress conditions. WT 4 males, 3 females; cKO 4 males, 3 females.


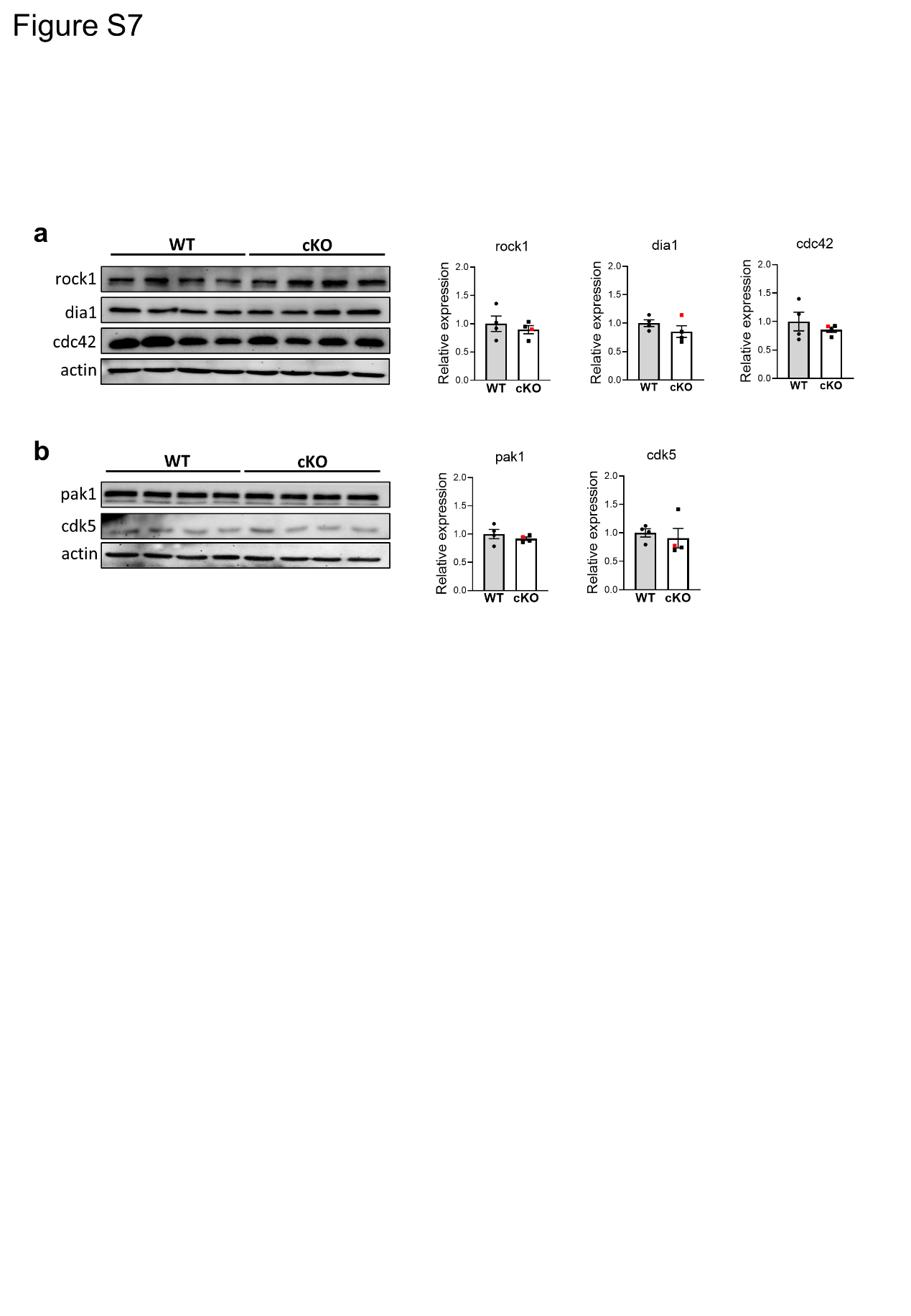


**Fig. S7.** **Expression of Rho GTPase–related signaling proteins in *Arhgap39* cKO mice.**

(**a**) Representative Western blot and quantification of Rock1 (t_(6)_ = 0.683, *p* = 0.519), Dia1 (t_(6)_ = 1.244, *p* = 0.26), and Cdc42 (t_(6)_ = 0.845, *p* = 0.43) protein levels in the hippocampus of WT and *Arhgap39* cKO mice. (**b**) Representative Western blot and quantification of Pak1 (t_(6)_ = 0.932, *p* = 0.387) and Cdk5 (t_(6)_ = 0.502, *p* = 0.633) protein levels in the hippocampus of WT and *Arhgap39* cKO mice. β-Actin was used as a loading control. WT 4 males; cKO 3 males, 1 female.


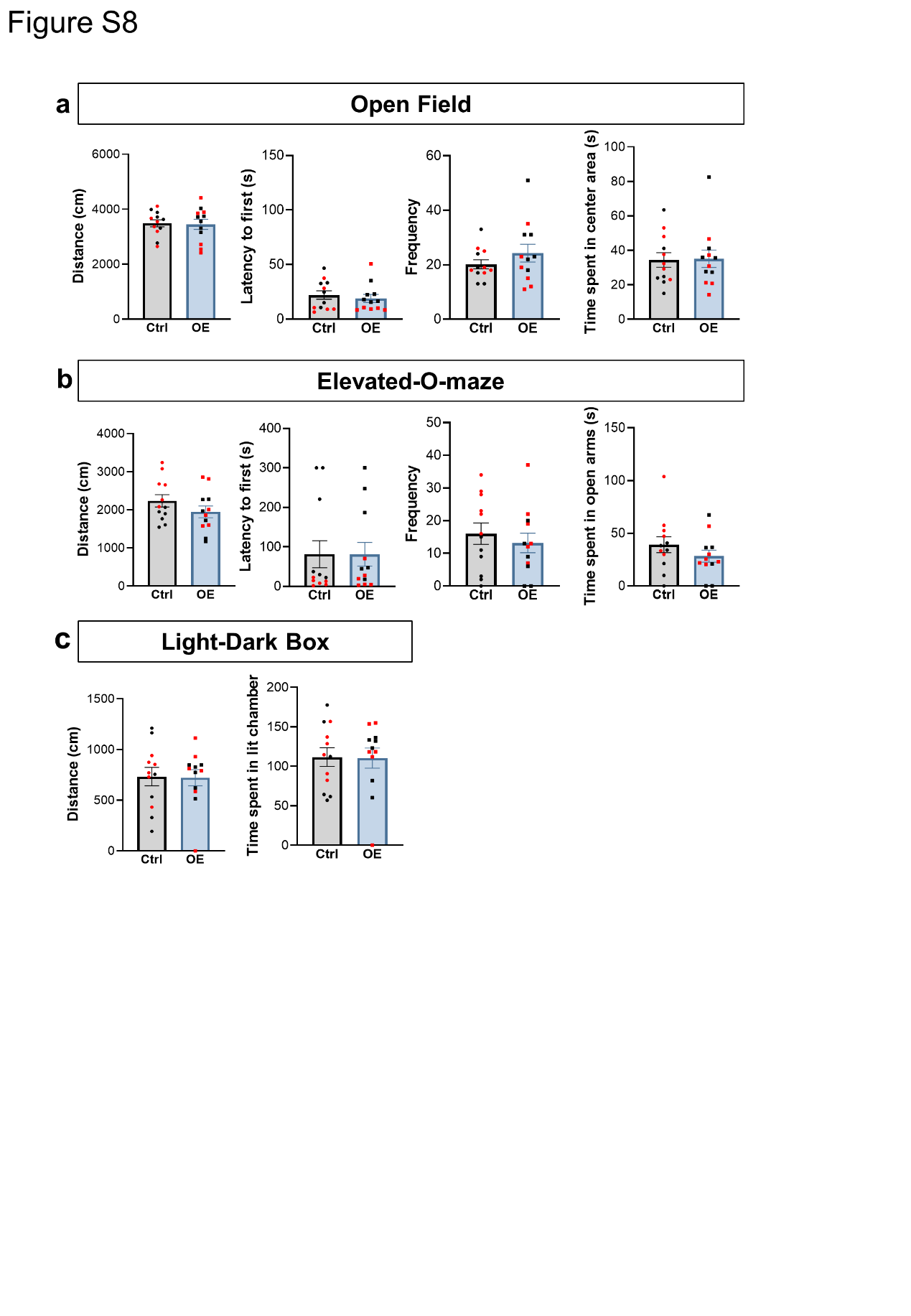


**Fig. S8. No anxiolytic effect of *Arhgap39* OE mice under basal conditions**

Performance of *Arhgap39* OE mice in (**a**) Open field test: total distance traveled (t_(22)_ = 0.178, *p* = 0.859), latency to first enter the center (t_(22)_ = 0.561, *p* = 0.58), number of center entries (t_(22)_ = 1.112, *p* = 0.278), and time spent in the center zone (t_(22)_ = 0.1, *p* = 0.92) were analyzed. (**b**) Elevated-O-Maze test: total distance traveled (t_(22)_ = 1.287, *p* = 0.211), latency to first enter the open arms (t_(22)_ = 0.001, *p* = 0.998), number of open arm entries (t_(22)_ = 0.638, *p* = 0.529), and time spent in the open arms (t_(22)_ = 1.143, *p* = 0.265) were analyzed. (**c**) Light-Dark box test: distance traveled in light compartment (t_(22)_ = 0.085, *p* = 0.932) and time spent in light compartment (t_(22)_ = 0.071, *p* = 0.943) were analyzed. WT 6 males, 6 females, cKO 6 males, 6 females.
